# Patch Test Results With the European Baseline Series, 2021/2022—Joint European Results of the ESSCA^A^ and the EBS^B^ Working Groups of the ESCD, and the GEIDAC^C^

**DOI:** 10.1111/cod.70134

**Published:** 2026-03-12

**Authors:** Wolfgang Uter, S. Mark Wilkinson, Olivier Aerts, Andrea Bauer, Leopoldo Borrego, Richard Brans, Timo Buhl, Heinrich Dickel, Aleksandra Dugonik, Francesca Larese Filon, Ana Giménez Arnau, Cataldo Patruno, Györgyi Pónyai, Stamatis Gregoriou, Steffen Schubert, Dagmar Simon, Luca Stingeni, Skaidra Valiukevičienė, Elke Weisshaar, Thomas Werfel, Margarida Gonçalo, Marie‐L. A. Schuttelaar, ESSCA and EBS ESCD Working Groups, and the GEIDAC, Birger Kränke, Alexander Navarini, Philip Spring, Ulrike Beiteke, Cecilia Dietrich, Sibylle Schliemann, Detlef Becker, Swen Malte John, Nicola Wagner, Dimitra Koumaki, Maddalena Napolitano, Anna Belloni Fortina, Francesca Larese Filon, Rosella Gallo, Thomas Rustemeyer, Radoslaw Spiewak, Maja Kalac Pandurovic, Tanja Kmecl, Mojca Simončič Godnič, Nada Kecelj, Philippa Cousen, Ella Dendooven, Juan Francisco Silvestre Salvador, José Manuel Carrascosa Carrillo, Mónica Munera Campos, Esther Serra Baldrich, Gemma Melé‐Ninot, Cristina Barrabés Torrella, David Pesqué, María Antonia Pastor Nieto, Francisco Navarro Triviño, Inmaculada Ruiz Gonzales, Enrique Gómez de la Fuente, Susana Córdoba Guijarro, Francisco Javier Ortiz de Frutos, Fátima Tous Romero, Tatiana Sanz Sánchez, Pablo Chicharro Manso, Marta Andreu Barasoain, Marta Elosua González, Araceli Sánchez Gilo, Paloma Sánchez‐Pedreño Guillén, Pedro Mercader‐García, Carmen Paredes Suárez, María Elena Gatica Ortega, José Juan Pereyra Rodríguez, Javier Miquel Miquel, Mercedes Rodríguez Serna, Violeta Zaragoza Ninet, Ricardo González Pérez

**Affiliations:** ^1^ Department of Medical Informatics, Biometry and Epidemiology Friedrich‐Alexander‐Universität Erlangen‐Nürnberg Erlangen Germany; ^2^ Dermatology Department Leeds Teaching Hospitals NHS Trust Leeds UK; ^3^ Department of Dermatology University Hospital Antwerp (UZA) and Research Group Immunology, Infla‐Med Centre of Excellence, University of Antwerp Antwerp Belgium; ^4^ Department of Dermatology University Hospital Carl Gustav Carus, Technical University Dresden Germany; ^5^ Complejo Hospitalario Universitario Insular Materno Infantil, Universidad de Las Palmas de Gran Canaria Las Palmas de Gran Canaria Spain; ^6^ Institute for Interdisciplinary Dermatologic Prevention and Rehabilitation (iDerm) at the Osnabrück University Osnabrück Germany; ^7^ Department of Dermatology, Venereology and Allergology University Medical Center Göttingen Göttingen Germany; ^8^ Department of Dermatology, Venereology and Allergology St. Josef Hospital, University Medical Center, Ruhr University Bochum Bochum Germany; ^9^ Department of Dermatology University Medical Centre Maribor Maribor Slovenia; ^10^ Department of Medical, Surgical and Health Sciences, Occupational Medicine University of Trieste Trieste Italy; ^11^ Department of Dermatology Hospital del Mar, Research Institute, Universitat Pompeu Fabra de Barcelona Barcelona Spain; ^12^ Department of Medicine and Health Sciences “Vincenzo Tiberio” University of Molise Campobasso Italy; ^13^ Department of Dermatology, Venerology and Dermato‐Oncology Semmelweis University Budapest Hungary; ^14^ Department of Dermatology, Faculty of Medicine National and Kapodistrian University of Athens Athens Greece; ^15^ Information Network of Departments of Dermatology (IVDK) Institute at the Georg‐August University Göttingen, University Medical Centre Göttingen Göttingen Germany; ^16^ Department of Dermatology Inselspital, Bern University Hospital, University of Bern Bern Switzerland; ^17^ Dermatology Section, Department of Medicine and Surgery University of Perugia Perugia Italy; ^18^ Department of Skin and Venereal Diseases The Hospital of Lithuanian University of Health Sciences Kauno Klinikos Kaunas Lithuania; ^19^ Occupational Dermatology, Department of Dermatology University Hospital Heidelberg Heidelberg Germany; ^20^ Department of Dermatology and Allergy Hannover Medical School Hannover Germany; ^21^ Department of Dermatology University Hospital and Faculty of Medicine, University of Coimbra Coimbra Portugal; ^22^ Department of Dermatology University Medical Center Groningen, University of Groningen Groningen The Netherlands

**Keywords:** baseline series, clinical epidemiology, contact allergy, patch testing, RRID:SCR_001905, surveillance

## Abstract

**Background:**

Patch test results obtained with the European Baseline Series (EBS) in its current version serve both contact allergy surveillance and (re‐)assessing the diagnostic value of EBS allergens.

**Objectives:**

To present results of current EBS patch testing, obtained in 59 departments in 14 European countries during 2021 and 2022.

**Methods:**

Anonymised or pseudonymised individual data, and partly aggregated results, on demographic/clinical characteristics and patch test results with the EBS were prospectively collected, centrally pooled, and retrospectively analysed.

**Results:**

In 2021 and 2022, 18 832 patients were patch tested with the EBS. Sensitization to nickel remained most common (18.85 (18.29–19.43)% positivity (95% confidence interval)). Fragrance mix I and *Myroxylon pereirae* resin yielded very similar results with 6.39 (6.04–6.76)% and 6.5 (6.15–6.87)% positivity, respectively. Concerning preservatives, methylchloroisothiazolinone/methylisothiazolinone (MCI/MI) 0.02% aq. yielded 5.52 (5.11–5.96)% and MI 0.2% aq. yielded 5.28 (4.94–5.64)% positives. Testing formaldehyde 2% aq. identified almost one percentage point more positive reactions than 1% aq. (2.05 (1.81–2.32)% vs. 1.22 (0.99–1.48)). Positive reactions to the recently added allergens were most frequently seen to propolis (5.47 (5.12–5.84)%) and 2‐hydroxyethyl methacrylate (3.63 (3.32–3.96)%).

**Conclusions:**

Compared to the previous reporting period, surveillance results with the EBS were mostly stable. The results regarding Quaternium 15 (0.4 (0.29–0.53)% positives) justified its exclusion from the 2023 EBS version.

## 1 Introduction

A “baseline” series (BS) for patch testing should comprise those contact allergens which are of greatest importance and relevance for the majority of patients. It is thus regularly patch tested in all patients in whom allergic contact dermatitis is suspected or should be excluded. Only if the BS had already and validly been tested elsewhere within a certain time period before a current consultation, the BS is not tested again in some tertiary referral centres to avoid repetition. The set of allergens included in a BS should be adapted to changes of exposure, which may be seen over time and which may differ between geographical regions. Following an initial publication delineating the objectives of a BS [1], the criteria for inclusion have recently been revisited [2].

The European Baseline Series (EBS) for patch testing is usually modified by national contact dermatitis groups or single departments in terms of additions or omissions; hence, different numbers of patients tested will result for each allergen. This also concerns EBS allergens which are not mirrored in the TRUE Test (SmartPractice, Phoenix, AZ), a commercial patch test system with pre‐filled chambers, for example test preparations such as fragrance mix (FM) II or methylisothiazolinone, which are usually supplemented by those departments otherwise relying on the TRUE Test. Moreover, test concentrations of some EBS allergens may differ between departments depending on national availability of commercial test preparations and market authorizations [3].

The present paper summarises results obtained with the EBS in the years 2021/2022 by members of the European Surveillance System on Contact Allergies (ESSCA), a working group of the European Society of Contact Dermatitis (ESCD; https://www.escd.org), by members of the EBS taskforce of the ESCD, and by the Spanish “Grupo Español de Investigación en Dermatitis de Contacto y Alergia Cutánea” (GEIDAC)/“Registro Español de Dermatitis de Contacto” (REIDAC) surveillance network described in [4]. The 2019 version of the European Baseline Series (EBS) [5] has been used until end of 2022, followed by the 2023 version [6], with probably some adaptation period. Thus, the 2019 EBS version was valid during the present reporting period.

In addition to the EBS in a strict sense reported on here, several departments prospectively tested the recommended additions in consecutive patients. Results on these “audit allergens” will be published separately [7]. The objective of this article is to present patch test results with the EBS obtained in the years 2021/2022 from a wide spread of European departments with a view to informing a further revision of the EBS. TRUE Test results are also presented in this paper (in total), as the TRUE Test corresponds in a large part to the EBS. Thereby, similar reports on previous EBS results are continued and possible trends discussed; regarding the last three periods, see [8, 9, 10].

## 2 Methods

The ESSCA uses electronically collected patch test results along with a core set of clinical and demographic information as implemented by single departments or national contact dermatitis groups [11, 12, 13]. These include the SIDAPA (Società Italiana di Dermatologia Allergologica Professionale e Ambientale), the DKG (German Contact Dermatitis Research Group) in collaboration with the IVDK (Information Network of Departments of Dermatology), including Austrian and Swiss members, and the Slovenian contact dermatitis network. Standardisation of patch test procedures is achieved by adherence to the ESCD patch test guideline [14]. The present analysis combines actual data as well as aggregated data (results) covering the period 01/2021 to 12/2022 from three different sources, which are briefly described in the next paragraph. For further detail, see [10]. As patch test outcome, the maximum reaction between day (D) 3 and D5 (inclusive) was used, following current ESSCA practice (see discussion).

Generally, contributing departments submit either all patch test results or just patch test results obtained with the EBS (or national or local adaptations thereof) to the data centre in Erlangen. Information also includes important demographic and clinical information, ranging from “MOAHLFA(P) index” [15] characteristics as minimum to a wider scope of information according to the ESSCA “minimal dataset” definition [11, 16]. The three main sources include:

- Data from contributing departments delivered as an anonymous export, or partly in a pseudonymized format, where the pseudonym cannot be related to actual personal data except back in the contributing department itself, following national network standards.
- Members of the EBS working group contributing data either via a local academic implementation of a SoSci server (https://www.soscisurvey.de/), that is, an online platform for data entry, via spreadsheets, or as export from local hospital information systems.
- Aggregated data, that is results per allergen, partly also stratified for gender or age group, supplied by the members of the Spanish Contact Dermatitis and Skin Allergy Research Group (GEIDAC), as collected prospectively in the Spanish Contact Dermatitis Registry (REIDAC) using the OpenClinica platform (OpenClinica LLC and collaborators) until September 2022. Since then, the REIDAC is hosted on a RedCap platform located at the Research Unit of the Spanish Academy of Dermatology and Venerology [17]. Moreover, one UK department (Leeds) contributes such aggregated results data.

In the ESSCA data centre, data management and analysis was performed with the R software package (https://www.r‐project.org; RRID:SCR_001905), version 4.4.x., following pertinent guidelines [18]. For the calculation of 95% confidence intervals (CIs) to zero proportions an approximation to an exact CI was used [19]. Cross‐reactivity between contact allergens was illustrated by use of a bivariate odds ratio (OR) with accompanying 95% CI. On the level of countries, cross‐reactivity was analysed as ecological correlation and (adjusted) linear regression analysis, respectively, involving the % positives to a pair of allergens considered, as taken from the descriptive results. The latter type of analysis was adjusted for the a priori likelihood of a positive test result to a baseline series allergen as represented by the “P‐measure” [20].

## 3 Results

Among the 22 891 included patients consulting in the reporting period, 18 832 patients were patch tested with the EBS in 2021/22 with its 2019 version [2], possibly including consecutively tested additions later included in the 2023 version [6]. It should be noted that, for different reasons, several allergens included in the EBS have been tested in concentrations different from those indicated [2], that is, methylchloroisothiazolinone (MCI)/methylisothiazolinone (MI) 3:1 at 0.01% aq., MI at 0.05% aq., formaldehyde at 1% aq., methyldibromo glutaronitrile (MDBGN) at 0.3% pet., budesonide at 0.1% pet, and tixocortol pivalate at 1.0% pet. Further, the 3‐component mercapto mix 1% pet. has been tested in 5911 patients, and the 4‐component mix 2% pet., containing mercaptobenzothiazole, in 9799. These results are presented separately. Further 2702 patients were tested with the TRUE Test in Spain; in 340 both the TRUE Test and the EBS were tested in Spain, besides supplementing EBS allergens such as MI, formaldehyde 2% aq., FM II, hydroxyisohexyl 3‐cyclohexene carboxaldehyde (HICC), 2‐hydroxyethyl methacrylate (HEMA), textile dye mix, decyl glucoside, and propolis in patients otherwise tested just with the TRUE Test [17]. Thus, 21 194 patients had been tested with either the EBS and/or the TRUE Test. The individual contribution by country and department is shown in Table S1. Population characteristics according to the MOAHLFA index [21], extended by the “P‐measure”, that is, the proportion of patients positive to at least one allergen from the EBS [15, 20], are illustrated in Table 1.

**TABLE 1:** Demographic and clinical characteristics according to the MOAHLFA(P) index [15] and number of patients tested with the European baseline series (including the TRUE Test baseline series in Spain, see Table 3) per country.

Country	*N* (tested)	M	O	A	H	L	F	A^(2)^	P
AT	184	19.7	14.9	20.2	23.4	4.3	16.5	63.8	52.7
BE	891	29.2	10.1	37.7	19.6	7	25.7	56.9	51.1
CH	1130	35.2	15.9	23.2	25.3	7.1	16.3	66.2	46.9
DE	3553	35.5	31.7	28.3	41.7	4.3	9.2	71.3	30.4
ES	5227	31.2	8.5	19	31.7	4.8	21.7	66	36.1
GR	499	29.9	27.9	18.2	33.3	5.2	23.4	60.1	66.7
HU	509	24.2	0	15.7	39.7	2.8	20.4	63.3	50.7
IT	2090	28.7	4.8	15.9	31	1.4	4.5	57.1	30.3
LT	701	23.4	22	7.7	30.4	7.3	25.1	46.6	30.5
NL	1973	31.1	10.6	31.4	11.1	2.4	5.2	60.8	55.8
PL	85	25.9	0	20	0	0	0	31.8	60
PT	526	34.8	21.9	15.8	32.9	3	18.3	65.6	44.3
SI	1493	29.2	1	5.4	42.5	14.7	24.7	59.1	42.9
UK	2333	29.8	5.1	49.1	26	4.5	37.6	46.8	49.7
Total	21 194	31	12.8	24.2	30.7	5	18.1	61.5	40.9

*Note*: M, % male patients; O, % patients with occupational dermatitis; A, % patients with atopic dermatitis; H, % patients with hand dermatitis; L, % patients with leg dermatitis; F, % patients with face dermatitis; A^(2)^, % patients age 40 and above; P, share of patients with at least one positive reaction to a baseline series allergen.

Patch test results with the EBS are shown in Table 2, grouped for allergen classes. A supplemental analysis stratified for the contributing countries can be found in Table S2. As the composition of the TRUE Test (panels 1 to 3) partly differs from the EBS, and as concentrations (effectively: doses per area) are partly different, the results obtained in the Spanish and (selectively) one Dutch department are presented separately in Table 3.

**TABLE 2:** Patch test results (day 3 to day 5) with the European Baseline Series in consecutive patients in the 59 active departments of the European Surveillance System on Contact Allergies (ESSCA), additional contributors from the EBS working group, and the contributing “Grupo Español de Investigación en Dermatitis de Contacto y Alergia Cutánea” (GEIDAC) members (TRUE Test results see Table 3).

Allergen	Conc. (%)/mg/cm^2^	Tested	+/++/+++	?+/IR	% pos. (95% CI)
Metals					
Potassium dichromate	0.5/0.2	18 048	784	384	4.34 (4.05–4.65)
Cobalt chloride	1.0/0.4	18 112	1251	484	6.91 (6.54–7.29)
Nickel sulphate	5.0/2.0	18 310	3452	351	18.85 (18.29–19.43)
Fragrances					
Fragrance mix I	8.0/3.2	17 999	1150	258	6.39 (6.04–6.76)
Fragrance mix II	14/5.6	20 324	737	228	3.63 (3.37–3.89)
HICC	5.0/2.0	16 805	159	35	0.95 (0.81–1.1)
*Myroxylon pereirae* resin (balsam of Peru)	25/10	18 282	1189	357	6.5 (6.15–6.87)
Preservatives					
Formaldehyde	1.0^a^/0.3	8148	99	69	1.22 (0.99–1.48)
Formaldehyde	2.0^a^/0.6	12 264	252	56	2.05 (1.81–2.32)
MCI/MI 3:1	0.01^a^/0.003	9172	314	41	3.42 (3.06–3.82)
MCI/MI 3:1	0.02^a^/0.006	11 406	630	117	5.52 (5.11–5.96)
Methylisothiazolinone	0.05^a^/0.015	4365	151	21	3.46 (2.94–4.04)
Methylisothiazolinone	0.20^a^/0.06	16 201	856	145	5.28 (4.94–5.64)
Paraben mix	16/6.4	8405	68	74	0.81 (0.63–1.02)
Quaternium‐15	1.0/0.4	11 473	46	12	0.4 (0.29–0.53)
Methyldibromo glutaronitrile	0.3/0.12	4673	196	111	4.19 (3.64–4.81)
Methyldibromo glutaronitrile	0.5/0.2	7874	418	103	5.31 (4.82–5.83)
Medicaments, excipients					
Caine mix III (Benzo‐,Cincho‐,Tetracaine)	10/4.0	7912	117	54	1.48 (1.22–1.77)
Budesonide	0.01/0.004	10 642	81	45	0.76 (0.6–0.95)
Budesonide	0.1/0.04	2854	3	4	0.11 (0.02–0.31)
Tixocortol pivalate	0.1/0.04	8337	41	23	0.49 (0.35–0.67)
Tixocortol pivalate	1.0/0.4	3715	14	15	0.38 (0.21–0.63)
Neomycin sulphate	20/8.0	13 486	104	15	0.77 (0.63–0.93)
Lanolin (wool alcohols)	30/12	18 135	253	108	1.4 (1.23–1.58)
Rubber additives					
Thiuram mix	1.0/0.4	18 217	473	48	2.6 (2.37–2.84)
*N*‐Isopropyl‐*N*′‐phenyl‐*p*‐phenylenediamine	0.1/0.04	17 805	99	44	0.56 (0.45–0.68)
Mercapto mix^(i)^	1.0/0.4	5911	33	22	0.56 (0.38–0.78)
Mercapto mix^(ii)^	2.0/0.8	9799	32	6	0.33 (0.22–0.46)
Mercaptobenzothiazole	2.0/0.8	18 324	84	37	0.46 (0.37–0.57)
Resins/glues					
Colophonium	20/8.0	18 132	535	54	2.95 (2.71–3.21)
Epoxy resin	1.0/0.4	16 615	197	30	1.19 (1.03–1.36)
*p*‐*tert*‐Butylphenol formaldehyde resin	1.0/0.4	13 558	77	34	0.57 (0.45–0.71)
2‐Hydroxyethyl methacrylate	2.0/0.8	13 472	489	33	3.63 (3.32–3.96)
Other					
*p*‐Phenylenediamine	1.0/0.4	13 563	522	20	3.85 (3.53–4.19)
Sesquiterpene lactone mix	0.1/0.04	12 803	84	22	0.66 (0.52–0.81)
Propolis	10/4.0	15 700	859	279	5.47 (5.12–5.84)
Textile dye mix	6.6/2.64	10 713	406	108	3.79 (3.44–4.17)

*Note*: Conc., concentration in %, tested in petrolatum, except where indicated otherwise: superscript a, aqua. For composition of mixes see [5]. Epoxy resin, diglycidyl ether of bisphenol A; HICC, hydroxyisohexyl 3‐cyclohexene carboxaldehyde; mercapto mix^(i)^, containing N‐cyclohexylbenzothiazyl sulfenamide, dibenzothiazyl disulfide, and morpholinylmercaptobenzothiazole; mercapto mix^(ii)^, containing N‐cyclohexylbenzothiazyl sulfenamide, mercaptobenzothiazole, dibenzothiazyl disulfide, and morpholinylmercaptobenzothiazole; MCI, methylchloroisothiazolinone; MI, methylisothiazolinone.

**TABLE 3:** Patch test results obtained with the TRUE Test used as baseline series (supplemented with other, pet.‐ or aq.‐based allergens included in Table 2) in 12 Spanish departments in *n* = 2702 patients.

Allergen	mg/cm^2^	+/++/+++	% pos. (95% CI)
Potassium dichromate	0.054	63	2.33 (1.8–2.97)
Cobalt (II)‐chloride 6*H_2_O	0.02	107	3.96 (3.26–4.77)
Nickel (II)‐sulphate hexahydrate	0.2	665	24.61 (23–26.28)
Gold sodium thiosulfate	0.075	165	6.11 (5.23–7.08)
Fragrance mix	0.5	103	3.81 (3.12–4.6)
Balsam of Peru (*Myroxolon pereirae*)	0.8	46	1.7 (1.25–2.26)
Formaldehyde	0.18	28	1.04 (0.69–1.49)
MCI/MI	0.004	147	5.44 (4.62–6.36)
Paraben mix	1	8	0.3 (0.13–0.58)
Quaternium 15	0.1	27	1 (0.66–1.45)
Diazolidinyl urea	0.55	13	0.48 (0.26–0.82)
Imidazolidinyl urea (Germall 115)	0.6	16	0.59 (0.34–0.96)
Thiomersal (Thimerosal)^a^	0.007	97	3.59 (2.92–4.36)
2‐Bromo‐2‐nitro‐1,3‐propanediol	0.25	28	1.04 (0.69–1.49)
Methyldibromo glutaronitrile	0.005	8	0.3 (0.13–0.58)
Caine Mix III (Benzo‐,Cincho‐,Tetracaine)	0.63	26	0.96 (0.63–1.41)
Budesonide	0.001	15	0.56 (0.31–0.91)
Tixocortol‐pivalate	0.002	8	0.3 (0.13–0.58)
Hydrocortisone‐17‐butyrate	0.02	10	0.37 (0.18–0.68)
Neomycin sulphate	0.6	17	0.63 (0.37–1.01)
Ethylene diamine‐di‐HCl^b^	0.05	32	1.18 (0.81–1.67)
Quinoline mix	0.19	3	0.11 (0.02–0.32)
Bacitracin	0.6	2	0.07 (0.01–0.27)
Lanolin (wool fat) alcohols	1	17	0.63 (0.37–1.01)
Thiuram mix	0.027	30	1.11 (0.75–1.58)
PPD (black rubber) mix^c^	0.075	24	0.89 (0.57–1.32)
Mercapto mix (CBS, MBTS, MOR)	0.075	10	0.37 (0.18–0.68)
Mercaptobenzothiazole	0.075	8	0.3 (0.13–0.58)
Carba mix^d^	0.25	47	1.74 (1.28–2.31)
Colophonium (Rosin)	1.2	29	1.07 (0.72–1.54)
Epoxy resin	0.05	32	1.18 (0.81–1.67)
*p*‐*tert*‐Butylphenol formaldehyde resin (PTBFR)	0.045	58	2.15 (1.63–2.77)
p‐Phenylenediamine (PPD)	0.08	107	3.96 (3.26–4.77)
Parthenolide	0.003	4	0.15 (0.04–0.38)

*Note*: TRUE Test had been tested in addition to the full EBS in Groningen in *n* = 158 patients; results only for those allergens which are not part of the EBS are shown in the footnote.

^a^ Thiomersal yielded 0 (0 (0–2.31)%) positive reactions.

^b^ Ethylene diamine‐di‐HCl yielded 1 (0.63 (0.02–3.48)%) positive reaction.

^c^ Black rubber mix yielded 1 (0.63 (0.02–3.48)%) positive reaction.

^d^ Carba mix yielded 3 (1.9 (0.39–5.45)%) positive reactions.

Positive reactions to nickel were observed most frequently (18.85 (18.29–19.43)% positivity (95% CI in parentheses)), see Table 2; the higher share seen with the TRUE Test (24.12 (22.56–25.73)%, Table 3) can probably be attributed to this being used by Spanish departments; however, also the Italian and Greek prevalences are high (Table S2). Among the fragrances and related substances tested in the EBS, FM I and *Myroxylon pereirae* resin (balsam of Peru) yielded very similar results with 6.39 (6.04–6.76)% and 6.5 (6.15–6.87)% pos., respectively. HICC was found positive in 0.95 (0.81–1.1)%. Concomitant reactivity between FM I and FM II was significant: OR 10.85 (95% CI: 8.97–13.12). The share of patients reacting to FM I or FM II (or both) was 1250/13511 (9.3%); if additionally *M. pereirae* resin is considered, the proportion of patients positive to at least one of these fragrance markers is 1853/13475 (13.8%). The correlation between positive reactions to FM I and FM II on the level of countries was strong (Spearman ρ= 0.62) and significant (*p* = 0.029). If, however, the “P‐measure” is considered as adjustment factor to represent the average likelihood to be diagnosed with some contact sensitization, this relationship becomes insignificant.

**FIGURE 1:**
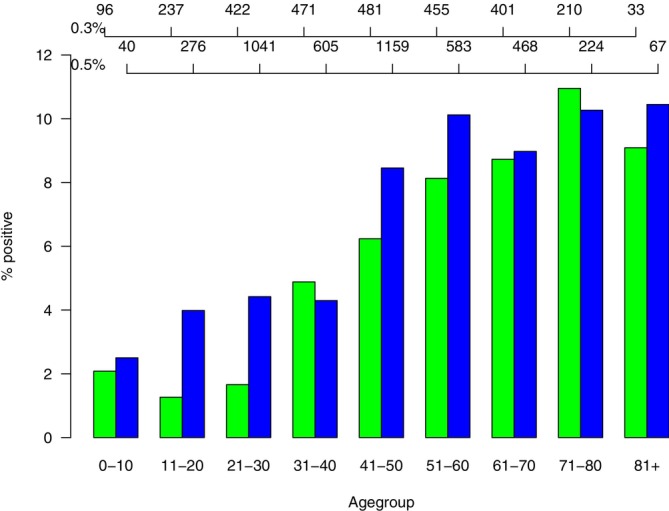
Age‐stratified % positive reactions to methyldibromo glutaronitrile (MDBGN) tested 0.3% pet. (green) and 0.5% pet. (blue) among patients tested in the ESSCA network, 2021–2022. Top x‐axes: Number of tested in each age group, top: MDBGN 0.3% pet., below: 0.5% pet.

Biocides (preservatives) comprise the largest group of allergens in the EBS (*n* = 6). Formaldehyde 2% aq. yielded somewhat less than one percentage point more positive reactions than the 1% concentration (2.05 (1.81–2.32)% vs. 1.22 (0.99–1.48)%). However, the results are difficult to compare, as they were obtained in different patients. The higher concentration of MCI/MI and MI alone, respectively, as recommended [2, 5], elicited positive reactions more frequently compared to the lower concentration still in use (Table 2), namely, in 5.52 (5.11–5.96)% to MCI/MI 0.02% aq. and 5.28 (4.94–5.64)% to MI 0.2% aq., respectively. MCI/MI tested in the TRUE Test yielded a result very similar to MCI/MI 0.02% aq. with 5.59 (4.78–6.5)% positive reactions (Table 3). While positive reactions to paraben mix and quaternium 15 were notoriously rare, the prevalence of MDBGN sensitization was still high, with overall 4.19 (3.64–4.81)% when tested 0.3% pet. and 5.31 (4.82–5.83)% when tested 0.5% pet., while only 0.33 (0.15–0.63)% positive reactions were observed when MDBGN 0.005 mg/cm^2^ was tested in Spain using the TRUE Test. If results with the two pet.‐based test preparations are stratified for age (Figure 1), a marked positive age‐gradient is evident; *p* < 0.001 (Cochrane‐Armitage trend test) in both cases. This is in contrast to formaldehyde (2% aq.) analysed in comparison, in which no trend whatsoever was found (*p* = 0.7).

Contact sensitization to rubber allergens beyond thiuram mix is not common, the frequency of positive reactions being below 1% in consecutively patch tested patients. Thiuram mix yielded 2.6 (2.37–2.84)% positive reactions when tested in the EBS and 1.19 (0.82–1.66)% using the TRUE Test. Combining reactivity to thiuram mix, mercaptobenzothiazole, and *N*‐isopropyl‐*N'*‐phenyl‐*p*‐phenylenediamine (IPPD), among the 13 848 patients tested with at least one of these rubber allergens, usually all three of them, 510 reacted positively. Among these latter patients, 270 suffered from hand dermatitis (crude OR: 2.74 (95% CI: 2.29–3.27)), thereof 148 with occupational contact dermatitis. Positive patch test reactions to colophonium, epoxy resin, and *p*‐*tert*‐butylphenol formaldehyde resin were seen in 2.95 (2.71–3.21)%, 1.19 (1.03–1.36)%, and 0.57 (0.45–0.71)%, respectively, and with the TRUE Test, in 1.22 (0.85–1.7)%, 1.33 (0.94–1.82)%, and 2.1 (1.6–2.69)%, respectively.

Positive reactions to the corticosteroid screening allergens and to neomycin sulfate were rare in both EBS and TRUE Test results. Caine mix III 10% pet. elicited 1.48 (1.22–1.77)% pos.; in the TRUE Test, the frequency of positive reactions was slightly lower at 0.98 (0.65–1.41)%. Patch tests with both dye‐related allergens, *p*‐phenylenediamine (PPD) 1% pet. and textile dye mix 6.6% pet., yielded very similar results, with 3.85 (3.53–4.19)% and 3.79 (3.44–4.17)% positive reactions, respectively (Table 2). Among 6899 patients simultaneously tested with both PPD and textile dye mix, coupled reactivity was marked, with 122 patients reacting to both, 113 only to PPD, and 162 only to textile dye mix (OR: 43.33 (95% CI: 32.11–58.47)).

Among the substances added to the EBS in 2019, propolis was most frequently positive with 5.47 (5.12–5.84)%, followed by HEMA at 3.63 (3.32–3.96)%, see Table 2. Coupled reactivity between propolis and *M. pereirae* resin was evaluated based on 10 535 patients tested with both; 167 patients reacted to both, 657 only to *M. pereirae* resin, and 544 only to propolis (OR: 4.28 (95% CI: 3.54–5.18)). Co‐reactivity between propolis and FM I was in a similar range, with 148 of 10 395 patients reacting to both allergens, 563 only to propolis, and 631 only to FM I (OR: 3.77 (95% CI: 3.09–4.6)). Concerning co‐reactivity of propolis with colophonium in 10 446 patients, 111 patients reacted to both natural mixtures, 582 only to propolis, and 282 only to colophonium (OR: 6.41 (95% CI: 5.07–8.11)).

## 4 Discussion

From collective experience during 2021 and especially 2022, we assume that the impact of the COVID‐19 pandemic on the work of patch test units has lessened, compared to the previous reporting period 2019/20, namely, affecting 2020. However, if comparing the contribution of single departments (Table S1) to the previous period [10], no clear pattern is discernible, and additional causes of variability in consultations among the contributing departments are likely. In the following, interesting aspects regarding the results seen in the different allergen groups shall be discussed, before addressing some limitations.

### 4.1 Metals

Positive reactions to nickel remain the most common; the higher prevalence in the TRUE‐Test is likely due to it being used almost exclusively in Spain, with a well‐known high nickel allergy prevalence compared to the European average [22]. Higher prevalences are generally observed in southern Europe (Spain, Italy, Portugal and Greece) [22, 23], but also in Austria and Poland. The latter countries are represented by just one department each, however, so that results may not be fully representative. Although nickel release from items in prolonged contact with skin has been regulated for over 20 years in the EU, the prevalence of nickel allergy remains high, prompting some authors to suggest revisions to the legal requirements for metallic products sold in the European market [24]. It has to be noted, however, that overall prevalence trends are not suited for assessing the effectiveness of regulation, as, owing to a cohort effect, sensitisation will still be diagnosed predominantly in women sensitised in the 60s to 70s; instead, age‐ and perhaps sex‐stratified trends and/or the share of currently relevant cases of contact allergy to nickel (see “Limitations” below) should ideally be addressed. While the prevalence of contact allergy to chromium is relatively stable at 4.34 (4.05–4.65)%, sensitization to cobalt has increased to 6.91 (6.54–7.29)% from 6.18% in 2019/2020 [10]. According to some authors cobalt sometimes even surpasses nickel sensitization, for example, in children with atopic dermatitis [25].

### 4.2 Fragrances

The EU has banned atranol, chloratranol, and HICC from cosmetics with the transition period ending in August 2021. Atranol and chloratranol are the most important sensitizers in *Evernia prunastri* (oakmoss absolute), which was the single ingredient of FM I most often positive in break‐down testing. Correspondingly, a significant decline of *E. prunastri* contact sensitization from 22.7% in 2012/13 to 16.3% in 2020/2021 was seen in an IVDK analysis of patients selectively tested with the FM I break‐down. During the same period, FM I crude sensitization prevalence declined from 10.4% to 5.0% [26]. Relating to previous ESSCA publications for comparison, FM I was positive in 7.4% (2009–2012), 7.8% (2013/2014), 5.8% (2015–2018), 6.8% (2019/2020), and is at 6.4% in the current reporting period (Table 2). Hence, in contrast to IVDK data, no comparable trend is discernible. This may be related to the wider catchment area of ESSCA patients, including regions in which sensitization prevalences to FM I are somewhat different [27], and/or putatively different patient selection particularly of the German patients (Table 1), and/or a somewhat greater potential for false‐negative readings in the IVDK (last reading being on D3, exceptionally D4), compared to ESSCA (maximum reaction of D3, D4, and D5).

HICC, which is one of the ingredients of FM II, is additionally tested separately in the EBS since the inception of FM II, as it was the most important allergen at the time [28]. In above IVDK data, a decline of FM II sensitization prevalence from 5.3% in 2012/2013 to 3.2% in 2020/2021 has been observed, in parallel to a decline of HICC sensitization prevalence from 2.0% to 1.2% [26]. ESSCA data showed no consistent pattern for FM II sensitization prevalence, with 4.04% (2009–2012), 4.0% (2013/2014), 3.26% (2015–2018), 3.77% (2019/2020) and presently 3.6% positives (Table 2). In contrast, HICC sensitization prevalence nearly halved from 1.79% in 2009–2012 to presently 0.9%, in line with a previous analysis focusing on the HICC sensitization trend [29]. The prevalence of HICC contact allergy is decreasing, diagnosed HICC contact allergy will be lacking current relevance more and more often, and not all, but at least a substantial share of HICC contact allergy is detected by FM II (followed by targeted break‐down testing of FM II constituents). Hence, it can be argued that HICC 5% pet. might be removed from the EBS [30], although others advocate maintaining HICC in the EBS to further monitor the effectiveness of the HICC ban [31] in cosmetic products and—linked by “horizontal legislation”—detergents [1], but not elsewhere.

The considerable variability of FM I and FM II sensitization prevalence between countries has been noted before, as recently reviewed [27]. The observed relatively strong correlation on the ecological level of countries between FM I and FM II contact sensitization (ρ = 0.62 rank correlation) seems, on first sight, to support a notion of differing fashion habits and thus sensitization risk. However, taking the general likelihood to be diagnosed with at least one contact allergy (also per country) as adjustment factor into account, this correlation becomes insignificant and is thus probably not specific.

Taking together reactivity to FM I, FM II and *M. pereirae* resin (balsam of Peru), the latter a “fragrance mix provided by nature”, to estimate the burden of fragrance allergy morbidity, it is evident that fragrances, despite preventive efforts outlined above, are still important allergens in patch tested patients in whom allergic contact dermatitis is suspected or should be excluded, yielding 13.8% positive reactions altogether. Although currently still tested as recommended additions, mainly owing to concerns regarding a high share of irritant or doubtful reactions seen [32], several researchers have stressed that additional testing of limonene and linalool hydroperoxides is important for the diagnosis of fragrance contact allergy, also in children [33, 34]. Sensitization to *M. pereirae* resin, although not used as such in cosmetics, is fairly stable and still worth testing as it allows detection of a remarkable number of relevant fragrance allergies [35].

It is hoped that (self‐regulatory) risk assessment based on combining evidence on (i) sensitisation hazard (potency) and (ii) typical exposure to cosmetic products which may contain fragrances, also in an aggregated form (i.e., through use of multiple scented products) termed “Quantitative Risk Assessment” (QRA, presently in its second version) will help reducing induction of sensitisation. However, QRA 2 is still in a process of evaluation [36], and a study intending to indicatively verify the usefulness of QRA 2 is presently under way, results being expected only in some years [37].

### 4.3 Biocides (Preservatives)

Formaldehyde is still a common sensitizer, albeit slightly less prevalent than before [10], and certainly when compared to the US [38]. Its hidden presence, especially in cosmetics [39], but also non‐cosmetic products such as sanitary napkins [40], is problematic. Sensitization to formaldehyde is detected in more patients using 2% aq. as test concentration, compared to 1% aq.; however, because different patients had been tested with the two preparations, no conclusion can be drawn based on the present data. Previous studies have, however, unequivocally proven the value of using formaldehyde 2% aq. in diagnosing patients with contact allergies to formaldehyde releasers [41], which play a much larger role than formaldehyde itself at least in cosmetic products. Application with a micropipette is necessary for correct dosing [42].

MDBGN sensitization still is high with both test concentrations used, similar to previous reports. The shortcomings of the TRUE Test MDBGN preparation have already been discussed [10]. In an attempt to elucidate whether the ban of MDBGN in cosmetics in 2007/08 could have had an effect on sensitization frequency, we age‐stratified results (Figure 1). This seemed to suggest a markedly lower prevalence of sensitization in persons up to 30 years (especially with the 0.3% concentration), which could be in line with some effect of this intervention. However, an analysis of data from St. John's Institute of Dermatology, Guy's Hospital, London, obtained 1985–2014, showed exactly the same pattern during a period where MDBGN appeared on the market, sensitization peaked, and MDBGN was banned in cosmetics [43]. Careful evaluation of clinical relevance in case of an allergic patch test reaction to MDBGN, especially in younger patients, may shed more light on the persisting problem of MDBGN exposure and contact sensitization [44]—while regarding older patients, it seems fair to assume that for an undetermined share of these, sensitization may be “historical”, that is, not relevant for current dermatitis. The striking differences between European countries, with high prevalences of MDBGN contact allergy seen in Greece, Belgium, and The Netherlands, and low prevalences in others, need explanation.

Concerning MCI/MI contact allergy, the present sensitization prevalence almost reached pre‐epidemic levels: Compared to ESSCA results covering 2009–2012 [45], when MCI/MI 0.01% aq. elicited 3.9%, and MCI/MI 0.02% aq. 4.4% positive reactions, the current figures are similar for MCI/MI 0.01% (3.4%) and still slightly higher for MCI/MI 0.02% (5.5%). Regarding MI itself, no stable estimates are available, as MI had just been included into the EBS towards the end of the former study period. At any rate, a recent analysis comparing MCI/MI and MI sensitization trends between Europe and North America has unequivocally proven the beneficial effect of regulatory action taken in Europe, limiting exposure to MI by cosmetic products, in terms of containing that unprecedented contact allergy epidemic [46]. Currently, however, increasing exposure and sensitization from non‐cosmetic sources is of concern [47]. Recently reported examples include the continued presence of MI in water‐based paints [48], ironing waters [49], nitrile gloves [50], dog cosmetics [51], and even pharmaceuticals (e.g., haemorrhoidal creams) [52] and medical devices, for example ultrasound gel [53].

Finally, parabens, although the share of positive reactions increased slightly to 0.81 (0.63–1.02)% as opposed to previous data (0.56%) [10], remain rare sensitizers. The debate continues whether paraben mix should possibly be removed from the EBS, as most cases of sensitization are related to topical medications and less frequently to cosmetics; hence, it might be considered to shift them to specialised series [54].

### 4.4 Rubber, Plastic, and Glues

Thiuram mix still is the most important rubber allergen in the EBS. This result is in line with that of a recent Danish single centre study reporting a continuously high prevalence of positive patch test reactions to thiuram mix in consecutively tested patients over time [55]. This observation is remarkable, as at least nowadays dithiocarbamates are by far the most commonly used accelerators in rubber gloves [56], whereas thiurams are hardly found. However, the thiurams included in the mix can also represent the corresponding dithiocarbamates, as both form a redox pair, that is, transforming into each other by oxidation or reduction. For instance, one molecule of diethylthiuram disulfide will cleave to two molecules of diethyl dithiocarbamate (as acid). Moreover, cross‐reactivity has recently been confirmed experimentally in a mouse model [57]. Hence, it has been argued that thiurams may actually be better screening substances for dithiocarbamate contact allergy than the dithiocarbamates themselves [58].

By contrast, sensitization prevalences observed with other rubber compounds in the EBS, that is, the benzothiazoles as well as IPPD, are low. Although difficult to compare, the yield with IPPD 0.1% pet. in the EBS, with 0.56 (0.45–0.68)% positive reactions is markedly, if not significantly, lower than with black rubber mix 75 μg/cm^2^ included in the TRUE Test, eliciting 0.91 (0.59–1.33)% positive reactions. Previous results seem to indicate that patch testing with IPPD alone will under‐diagnose contact allergy to this class of antidegradants [58, 59]. The discussion of the best patch testing strategy for benzothiazoles—mercaptobenzothiazole (MBT) together with either the four‐component mercapto mix 2% pet. or, preferably, the three‐component mercapto mix 1% pet [58, 60] – shall not be re‐iterated here. A discussion whether rubber allergens beyond thiuram mix qualify for inclusion in the EBS appears warranted. However, the identification of rubber contact allergy being unsuspected from the patient history would be one reason to keep a minimum set of rubber allergens in the EBS, despite low prevalence. Furthermore, the fact that these allergens are often affecting patients with hand dermatitis, which may be chronic and often work‐related, would favour continued inclusion in the EBS. Lastly, a negative patch test result can largely rule out contact allergy to the vulcanizing allergens such tested and hence help patient counselling, particularly when selecting personal protective equipment.

Colophonium, with 2.95 (2.71–3.21)% sensitization prevalence, remains a ubiquitous skin sensitizer. According to recent data, children and adolescents are more often found sensitised [61], as well as females [62]. Many hidden exposures exist [63], well‐known sources may still be relevant (such as shoe glues) [64], and new exposures have been identified, including bees wax wraps promoted as eco‐friendly food wrap [65]. Particular concerns have been recently expressed concerning severe colophonium allergies from its presence in tapes used by athletes [66] and military personnel [67], in resin‐containing wound creams [68], and in medical devices used by diabetes patients [69, 70]. In the latter product type often modified colophonium derivatives are used, and contact allergy from these is not often detected by testing colophonium as included in the EBS.

The proportion of patients reacting to epoxy resin remains stable at 1.19 (1.03–1.36)% (TRUE Test 1.33 (0.94–1.82)%). Epoxy resin is typically recognised as an occupational sensitizer [71], sometimes leading to severe reactions [72]. Contact allergy is more frequently observed among male construction workers and painters [62, 73, 74]. However, reports indicate the emergence of epoxy resin contact allergy in females and children, often presenting with angioedema‐like symptoms, mostly due to recreational exposures [75, 76, 77].

The prevalence of sensitization to HEMA, which is considered a marker for sensitization also to other (meth)acrylates [78], is still rising from 2.32 (2.00–2.68)% in the period 2019/2020 [10], to 3.63 (3.32–3.96)% in 2021/2022. Most likely, this increase is related to increasing exposure to 2‐HEMA and other (meth)acrylates in nail cosmetics, despite the EU Cosmetics Regulation (EC 1223/2009) restricting the use of 2‐HEMA to nail cosmetics for professional use only since November 2020. Thus, the intended impact has not been achieved and further restrictions seem warranted [79]. This is further underscored by the fact that HEMA is by far the most common ingredient of nail cosmetics, found in nearly 60% of the products, and that violations of EU legislation such as lack of mandatory warnings regularly occur (in > 30% of nail cosmetics) [80]. Apart from nail cosmetics, 2‐HEMA and other (meth)acrylates are widely used in various applications, including dental materials, glues, coatings, paints, lacquers, printing ink, and bone cement, causing occupational and non‐occupational allergic contact dermatitis [81].

### 4.5 Medicaments, Excipients, Dyes, and Plant Materials

Following the last revision of the EBS in 2019, caine mix III 10% pet. has been included to replace benzocaine 5% pet. Of note, the share of positive reactions to caine mix III in the observation periods 2019/2020 (1.56 (1.28–1.89)% [10] and currently 1.48 (1.22–1.77)%) is two times higher than that to benzocaine observed 2015–2018 (0.69 (0.58–0.82)%). The sensitivity for detecting contact sensitization to benzocaine provided by this mix is acceptable, while cinchocaine and tetracaine contact allergy is only detected in a minority of those sensitised [82]. The corticosteroid markers in the EBS are altogether infrequent allergens, with, however, marked variation between countries and between the three test concentrations used, if the TRUE Test is additionally considered. The addition of supplementary corticosteroids, depending on national prescription habits, may increase the diagnostic yield [83]. This concept has recently been confirmed in Spain, where the addition of clobetasol propionate (0.1% in ethanol and 1% pet.; 23 positive) helped to diagnose 18/4338 additional patients as corticosteroid‐allergic who otherwise, relying on patch testing only tixocortol pivalate (9 positives) and budesonide (24 positives), would have been missed [84]. Confirmation in other countries (with perhaps differing prescription habits) and further refinement of the patch testing strategy with corticosteroids seems warranted.

Considering the broad use of lanolin (alcohols), a sensitization frequency of 1.4 (1.23–1.58)% is rather low but stable (2019/2020: 1.38 (1.20–1.58)%) [10]. In contrast to the US, the importance of neomycin sulphate as a contact allergen seems to be decreasing, for example from 1.24 (1.11–1.37)% in 2009–2012 [85], 1.23 (1.11–1.35)% in 2015–2018 [9], and 0.83 (0.67–1.01)% in 2019/2020 [10], to 0.77 (0.63–0.93)% at present. This steady decline has been linked to decreasing sales/prescriptions of neomycin‐containing products at least in Germany [85].

Contact allergy from disperse dyes is not uncommon and can be of particular importance in some patients, like those with atopic dermatitis or prurigo nodularis [86, 87]. Textile dye mix (TDM), currently at 6.6% in the EBS, yields a stable and high frequency of contact allergy (currently 3.79 (3.44–4.17)%, previously 3.58%, in 2019/2020 [10]), supporting its continued testing in consecutive patients. Nevertheless, to avoid potentially severe co‐reactions with *p*‐phenylenediamine (PPD), diagnosis would be more ideally made using a textile dye mix without Disperse Orange 3 (DO3) which is not yet commercially available, along with PPD as a surrogate for DO3. Recent Swedish and Danish data have shown that exclusion of DO3 from TDM is indeed feasible [2, 88].

Propolis sensitization appears to be rising compared to previous reports, with currently 5.47 (5.12–5.84)% positive patch test reactions, compared to 3.48 (3.16–3.82)% in 2019/2020 [10]. The association with FM I, *M. pereirae* resin or colophonium contact allergy was modest in the present analysis and considerably less than observed previously [89]. Given the rising popularity of “natural” cosmetic products and remedies, which often contain propolis, the growing prevalence of sensitisation to propolis may point to the need for further study of exposure sources and, eventually, preventive measures. Indeed in Germany, a correlation between propolis imports and use on the one hand, and propolis contact allergy on the other has been demonstrated [90]. However, there is evidence that variations in propolis composition exist, even among species of the genus Populus (poplar) and also depending on geographic origin [89]. The resulting heterogeneity of propolis as both used in patch test materials and consumer products [5] severely impacts diagnostic accuracy [91].

Sesquiterpene lactone mix (SLM), together with Compositae mix II (as recommended addition), continue to be important haptens to diagnose contact allergy from this plant family and also Lauraceae and Magnoliaceae [92, 93], although patch‐testing plants ‘as is’ with its extracts is equally of interest in selected patients [94]. The sensitization frequency to SLM is stable and low (0.66%); occasional increases may be explained by cross‐reactivity to isobornyl acrylate, a sensitizer found in diabetes devices [95].

### 4.6 Limitations

On a methodological line, as already mentioned in the preceding publication [10], ESSCA uses, since its inception in 1996, the maximum reaction between day 3 (D3) and D5 (inclusive) as a common denominator outcome accommodating the decisive reading result from a variety of reading schedules. However, a certain share of sensitised patients will exhibit a clear (be it weak) positive reaction only at a late reading such as at D7; therefore, the ESCD patch test guideline considers an “optimum” reading strategy including “D2 and D3 or D4 and around D7” readings [14]. By contrast, “D2 and D3 or, preferably, D4” readings which correspond to a large share of ESSCA data are regarded as just “acceptable” [14]. The ESSCA working group will discuss the evident advantage on the one hand, and the potential difficulties with employing a reading frame until D7 on the other hand, for example using the maximum reaction between D3 and D7. To just mention briefly one difficulty: departments reading only until D3, or D4 if no D3 reading is possible (like most IVDK departments) will have a downward bias in positive reading results compared to those departments who *regularly* (not in an aimed fashion based on some prior rationale or individually following‐up de‐novo doubtful reactions on D3 or D4) employ a D7 reading. This will most markedly affect, but not be limited to, allergens such as aminoglycosides or corticosteroids [96, 97].

Some well‐known characteristics of the TRUE Test, such as poor sensitivity of FM I [98] or particularly the severely under‐dosed MDBGN [10], and better detection of the MCI/MI preparation than with MCI/MI 100 ppm aq. [99] should be taken into account when comparing TRUE Test results with other results obtained using investigator‐loaded chambers and pet.‐/aq.‐based allergens. Again, the sensitization prevalence to MCI/MI 200 ppm aq. is very close to that obtained with the TRUE Test, so both can probably be regarded as equivalent, even though no direct comparison is possible.

Lastly, the present study did not analyse information on clinical relevance, as in the vast majority of previous ESSCA analyses. Thereby, the *current* importance of contact allergens cannot specifically be addressed; consideration of evidence on (changing) exposure provided by other information sources can only partially compensate for this. It would be desirable to implement a common assessment system for clinical relevance, which is both reliable to apply and yet detailed enough to enable meaningful insights. Examples include relatively simple systems like that employed by the North American Contact Dermatitis Group [100], the CODEX system [101], or—offering more information, but also more complexity—a proposal from the StanDerm (Horizon 2020 COST action TD‐1206) consortium [102].

## 5 Conclusion

Surveillance of the prevalence of contact sensitization to EBS allergens continues to provide insights into the current importance of contact allergens, contributing to maintaining an up‐to‐date baseline series for routine patch testing in Europe. Concerning the EBS, national adaptations and extensions based on typical geographical allergen exposures are useful; these added allergens are often an important starting point for co‐operative evaluation on a broader, European scale. Combined with similar surveillance results from other parts of the world, including but not limited to the USA and Canada [46], an even broader perspective, and further insights, of impacts of regulatory policies, but also occupational exposures and cultural practices on sensitisation risk can be obtained.

## Author Contributions

**Wolfgang Uter:** conceptualization, methodology, software, data curation, formal analysis, project administration, resources, writing – original draft, writing – review and editing. **S. Mark Wilkinson:** conceptualization, data curation, investigation, validation, resources, writing – original draft, writing – review and editing, project administration, supervision. **Olivier Aerts:** conceptualization, validation, investigation, writing – original draft, writing – review and editing, project administration, supervision. **Andrea Bauer:** investigation, validation, supervision, resources, writing – original draft, writing – review and editing. **Leopoldo Borrego:** conceptualization, methodology, software, data curation, investigation, validation, funding acquisition, writing – original draft, writing – review and editing, project administration. **Richard Brans:** conceptualization, methodology, data curation, supervision, validation, investigation, writing – original draft, writing – review and editing. **Timo Buhl:** conceptualization, validation, investigation, writing – review and editing, writing – original draft, supervision. **Heinrich Dickel:** conceptualization, validation, supervision, writing – original draft, writing – review and editing. **Aleksandra Dugonik:** methodology, software, data curation, writing – original draft, writing – review and editing, investigation, validation. **Francesca Larese Filon:** software, writing – review and editing, validation, investigation. **Ana Giménez Arnau:** investigation, supervision, writing – original draft, writing – review and editing, validation. **Cataldo Patruno:** investigation, writing – original draft, writing – review and editing, supervision. **Györgyi Pónyai:** investigation, validation, supervision, data curation, resources, writing – original draft, writing – review and editing. **Stamatis Gregoriou:** investigation, validation, supervision, resources, writing – original draft, writing – review and editing. **Steffen Schubert:** conceptualization, methodology, software, data curation, validation, supervision, resources, project administration, writing – original draft, writing – review and editing. **Dagmar Simon:** investigation, validation, supervision, writing – original draft, writing – review and editing. **Luca Stingeni:** conceptualization, software, data curation, supervision, validation, investigation, writing – original draft, writing – review and editing. **Skaidra Valiukevičienė:** investigation, validation, supervision, writing – original draft, writing – review and editing. **Elke Weisshaar:** writing – review and editing, investigation, validation, supervision. **Thomas Werfel:** investigation, validation, supervision, writing – review and editing. **Margarida Gonçalo:** conceptualization, writing – original draft, writing – review and editing, investigation, validation, supervision, data curation. **Marie‐L. A. Schuttelaar:** conceptualization, data curation, investigation, validation, supervision, resources, writing – original draft, writing – review and editing, funding acquisition.

## Funding

The REIDAC project is sponsored by the Spanish Academy of Dermatology and Venereology (Fundación Piel Sana) and has received funding from the Spanish Medicines and Health Products Agency (Agencia Española de Medicamentos y Productos Sanitarios; available at https://www.boe.es/boe/dias/2023/12/15/pdfs/BOE‐A‐2023‐25482.pdf), Sanofi‐Aventis, and LEO Pharma. The REIDAC sponsors were not involved in the proposal, preparation, design, data extraction, analysis, interpretation, drafting, editing, review, approval, or logistical support associated with this manuscript.

## Conflicts of Interest

W.U. receives research funds directed to the department from the cosmetic industry association IFRA. O.A. is investigator, consultant and/or speaker for LEO Pharma, Abbvie, Sanofi, L'Oréal/La Roche Posay, Novartis, Amgen, and Bioderma/NAOS. R.B. served as advisor and speaker for LEO Pharma. H.D. is investigator, consultant and/or speaker for Almirall Hermal, Stallergenes, LEO Pharma, Sanofi‐Aventis and Novartis Pharma. A.G.A. is or recently has been a speaker and/or advisor for and/or has received research funding from Almirall, Amgen, AstraZeneca, Avene, Blue ‐Print, Celldex, Escient Pharmaceutials, Genentech, GSK, Harmonic Bio, Incyte, Instituto Carlos III‐ FEDER, Jaspers, LEO Pharma, Menarini, Mitsubishi Tanabe Pharma, Noucor, Novartis, Sanofi–Regeneron, Septerna, Servier, Thermo Fisher Scientific, Uriach Pharma. M.G. has received honoraria for advisory boards and lectures from Novartis, Abbvie, Astra‐Zeneca, Almirall, Biocryst, LEO Pharma, Lilly, Pfizer, Sanofi and Takeda. C.P. is an advisor, consultant, speaker and/or investigator for AbbVie, Almirall, Amgen, Eli Lilly, Galderma, La Roche‐Posay, LEO Pharma, Novartis, Pfizer, Pierre Fabre, Sanofi. L.S. is an advisor, speaker and/or investigator for AbbVie, Almirall, BMS, Eli Lilly, LEO Pharma, Novartis, Pfizer and Sanofi. M.‐L.A.S. is an advisor, consultant, speaker and/or investigator for Abbvie, Pfizer, LEO Pharma, Regeneron, Sanofi‐Genzyme, Amgen, Incyte and Galderma, and has received research grants from Regeneron, Sanofi‐Genzyme, Novartis and Pfizer. The IVDK, maintained by the IVDK e.V., of which S.S. is an employee, is sponsored by the cosmetic and fragrance industry (associations) as well as by public funds. T.W. is an advisor, consultant, speaker and/or investigator for Abbvie, Pfizer, LEO Pharma, Novartis, Regeneron, Sanofi‐Genzyme, Eli Lilly and Galderma. S.M.W. has received travel reimbursement to attend meetings with the cosmetic industry. S.G. has been an advisor, consultant and speaker for Abbvie, Pfizer, Novartis, LEO Pharma, Menarini, Pierre Fabre, L'Oreal. The other authors declare no conflicts of interest.

## Supporting information


**Table S1:** Contribution by department and country, respectively. Additional contributions are from the “Grupo Español de Investigación en Dermatitis de Contacto y Alergia Cutánea” (GEIDAC):^6^ (i) additional 2865 patients tested with investigator‐loaded system, and (ii) 2702 using the TRUE Test in 2021/22.
**Table S2:** Patch test results (day 3 to day 5) with the European Baseline series, 2021–2022, stratified for country. Conc., concentration in %, all tested in pet., except where indicated otherwise: ^a^, aqua.

## Data Availability

The data are not publicly available due to privacy or ethical restrictions.

## Appendix A Collaborators

^A^ESSCA working group of the ESCD and ^B^EBS working group of the ESCD (members contributing data).

Birger Kränke, Department of Dermatology, Medical University of Graz, Graz, Austria, Graz, Austria (birger.kraenke@medunigraz.at); Olivier Aerts, Department of Dermatology, University Hospital Antwerp (UZA) and Research group Immunology, Infla‐Med Centre of Excellence, University of Antwerp, Antwerp, Belgium, Antwerp, Belgium (olivier.aerts@uza.be); Alexander Navarini, NA, Basel, Switzerland (alexander.navarini@usb.ch); Dagmar Simon, Department of Dermatology, Inselspital, Bern University Hospital, University of Bern, Bern, Switzerland, Bern, Switzerland (dagmar.simon@insel.ch); Philip Spring, Dermatologie et vénéréologie FMH, Center Médical d'Epalinges, Epalinges, Switzerland, Lausanne/Epalignes, Switzerland (springphil@hotmail.com); Ulrike Beiteke, Department of Dermatology, Dortmund, Germany, Dortmund, Germany (ulrike.beiteke@klinikumdo.de); Timo Buhl, Department of Dermatology, University Medicine, Göttingen, Germany, Göttingen, Germany (timo.buhl@med.uni‐goettingen.de); Cecilia Dietrich, Department of Dermatology, University of Schleswig‐Holstein, Campus Kiel, Kiel, Germany, Kiel, Germany (cdietrich@dermatology.uni‐kiel.de); Andrea Bauer, Department of Dermatology, University Hospital Carl Gustav Carus, Technical University of Dresden, Dresden, Germany, Dresden, Germany (Andrea.Bauer@uniklinikum‐dresden.de); Sibylle Schliemann, Department of Dermatology and Allergology, University Hospital Jena, Jena, Germany, Jena, Germany (schliemann@derma‐jena.de); Detlef Becker, Department of Dermatology, University of Mainz, Mainz, Germany, Mainz, Germany (detlef.becker@unimedizin‐mainz.de); Richard Brans, Institute for Interdisciplinary Dermatologic Prevention and Rehabilitation (iDerm), University of Osnabrück, Lower Saxony Institute for Occupational Dermatology (NIB), Osnabrück, Germany, Osnabrück, Germany (rbrans@uni‐osnabrueck.de); Swen Malte John, Institute for Interdisciplinary Dermatologic Prevention and Rehabilitation (iDerm), University of Osnabrück, Lower Saxony Institute for Occupational Dermatology (NIB), Osnabrück, Germany, Osnabrück, Germany (sjohn@uni‐osnabrueck.de); Nicola Wagner, Department of Dermatology, University of Erlangen‐Nürnberg, Erlangen, Germany, Erlangen, Germany (nicola.wagner@uk‐erlangen.de); Elke Weisshaar, Occupational Dermatology, Department of Dermatology, University Hospital Heidelberg, Heidelberg, Germany, Heidelberg, Germany (Elke.Weisshaar@med.uni‐heidelberg.de); Thomas Werfel, Medizinische Hochschule Hannover, Department of Immunodermatology and experimental Allergy, Hannover, Germany, Hannover, Germany (Werfel.Thomas@mh‐hannover.de); Heinrich Dickel, Department of Dermatology, Venereology and Allergology, Ruhr University Bochum, Bochum, Germany, Bochum, Germany (heinrich.dickel@ruhr‐uni‐bochum.de); Stamatis Gregoriou, National and Kapodistrian University of Athens, Faculty of Medicine, Andreas Sygros Hospital, Athens, Greece, Athens, Greece (stamgreg@yahoo.gr); Dimitra Koumaki, Department of Dermatology and Venereology, University Hospital of Heraklion, Heraklion, 71 110 Crete, Greece, Heraklion, Crete, Greece (dkoumaki@yahoo.gr); Györgyi Pónyai, National Institute for Dermatology and Venereology, Budapest, Hungary, Budapest, Hungary (gyorgyi.ponyai@gmail.com); Cataldo Patruno, Department of Medicine and Health Sciences “Vincenzo Tiberio”, University of Molise, Campobasso, Italy, Napoli, Italy (cataldo.patruno@unimol.it); Maddalena Napolitano, Department of Clinical Medicine and Surgery, University Federico II of Naples, Naples, Italy, Napoli, Italy (maddalena.napolitano@unina.it); Anna Belloni Fortina, Paediatric Dermatology Unit, Department of Medicine DIMED, University of Padova, Padova, Italy, Padova Paediatrica, Italy (anna.bellonifortina@unipd.it); Francesca Larese Filon, Department of Public Health, Occupational Medicine, University of Trieste, Trieste, Italy, Trieste, Italy (larese@units.it); Rosella Gallo, Clinica Dermatologica, IRCCS—AOU San Martino—IST and Department of Health Sciences, University of Genoa, 16132 Genoa, Italy, Genova, Italy (NA); Luca Stingeni, Dermatology Section Department of Medicine and Surgery—University of Perugia, Italy, Perugia, Italy (luca.stingeni@unipg.it); Skaidra Valiukevičienė, Department of Skin and Venereal Diseases, Lithuanian University of Health Sciences, Kaunas, Lithuania, Kaunas, Lithuania (skaidra.valiukeviciene@kaunoklinikos.lt); Marie‐Louise Schuttelaar, Department of Dermatology, University Medical Centre Groningen, University of Groningen, Groningen, The Netherlands, Groningen, The Netherlands (m.l.a.schuttelaar@umcg.nl); Thomas Rustemeyer, Department of Dermatology, Free University of Amsterdam, Amsterdam, The Netherlands, Amsterdam VU, The Netherlands (t.rustemeyer@amsterdamumc.nl); Radoslaw Spiewak, Department of Experimental Dermatology and Cosmetology, Faculty of Pharmacy, Jagiellonian University Medical College, Krakow, Poland, Krakow, Poland (spiewak.eu@gmail.com); Margarida Gonçalo, Clinic of Dermatology, University Hospital and Faculty of Medicine, University of Coimbra, Coimbra, Portugal, Coimbra, Portugal (mmgoncalo@gmail.com); Aleksandra Dugonik, Department of dermatology, University Medical Center Maribor, Maribor, Slovenia, Maribor/Univ., Slovenia (aleksandra.dugonik@ukc‐mb.si); Maja Kalac Pandurovic, Outpatient clinic of dermatology, Maribor, Maribor, Slovenia, Maribor/Clinic, Slovenia (maja.kp@derma‐mkp.si); Tanja Kmecl, Department of Dermatology, General Hospital Celje, Celje, Slovenia, Celje, Slovenia (tanja.kmecl@guest.arnes.si); Mojca Simončič Godnič, Department of dermatology, Novo mesto, (please edit), Slovenia, Novo Mesto, Slovenia (mojcasg78@gmail.com); Nada Kecelj, Outpatient clinc of dermatology, Ljubljana (please edit), Slovenia, Ljubljana/Clinic, Slovenia (nada.kecelj@kclj.si); Mark Wilkinson, Dermatology, Leeds Teaching Hospitals NHS Trust, Leeds, UK (s.m.wilkinson@btinternet.com); Philippa Cousen, James Cook University Hospital, Middlesbrough, UK (philippa.cousen@stees.nhs.uk); Ella Dendooven, University Hospital Antwerp (UZA) and Research group Immunology, Infla‐Med Centre of Excellence, University of Antwerp, Antwerp, Belgium (elladendooven@hotmail.com).

^C^GEIDAC (Members of the “Grupo Español de Investigación en Dermatitis de Contacto y Alergia Cutánea” [GEIDAC] contributing data to the present analysis [partly also listed as co‐author]).

Juan Francisco Silvestre Salvador (Dermatology Department, Hospital General Universitario de Alicante, Alicante, Spain); José Manuel Carrascosa Carrillo (Dermatology Department, Hospital Universitari Germans Trias i Pujol, Barcelona, Spain); Mónica Munera Campos, (Dermatology Department, Hospital Universitari Germans Trias i Pujol, Barcelona, Spain); Esther Serra Baldrich (Dermatology Department Hospital Universitario de La Santa Creu i Sant Pau, Barcelona, Spain); Gemma Melé‐Ninot (Dermatology Department, Hospital Universitari Sagrat Cor, Barcelona, Spain); Cristina Barrabés Torrella (Dermatology Department, Hospital Universitari Sagrat Cor, Barcelona, Spain); David Pesqué (Dermatology Department Hospital del Mar Research Institute, Barcelona, Spain); Ana María Giménez Arnau (Dermatology Department, Hospital del Mar Research Institute, Barcelona, Spain); María Antonia Pastor Nieto (Dermatology Department, Hospital Universitario de Guadalajara, Guadalajara, Spain); Francisco Navarro Triviño (Dermatology Department, Hospital Universitario San Cecilio, Granada, Spain); Leopoldo Borrego (Dermatology Department Complejo Hospitalario Universitario Insular Materno Infantil, Las Palmas de GC, Spain); Inmaculada Ruiz Gonzales (Dermatology Department, Complejo Asistencial Universitario de León, León, Spain); Enrique Gómez de la Fuente (Dermatology Department, Hospital Universitario Ramón y Cajal, Madrid, Spain); Susana Córdoba Guijarro (Dermatology Department, Hospital Universitario de Fuenlabrada, Madrid, Spain); Francisco Javier Ortiz de Frutos (Dermatology Department, Hospital Universitario Doce de Octubre, Madrid, Spain); Fátima Tous Romero (Dermatology Department, Hospital Universitario Doce de Octubre, Madrid, Spain); Tatiana Sanz Sánchez (Dermatology Department, Hospital Universitario Infanta Sofía, Madrid, Spain); Pablo Chicharro Manso (Dermatology Department, Hospital Universitario de la Princesa, Madrid, Spain); Marta Andreu Barasoain (Dermatology Department, Hospital Universitario Fundación de Alcorcón, Madrid, Spain); Marta Elosua González (Dermatology Department, Hospital Universitario Puerta de Hierro, Madrid, Spain); Araceli Sánchez Gilo (Dermatology Department, Hospital Universitario Rey Juan Carlos, Madrid, Spain); Paloma Sánchez‐Pedreño Guillén (Dermatology Department, Hospital Universitario Virgen de La Arrixaca, Murcia, Spain); Pedro Mercader‐García (Dermatology Department, Hospital General Universitario Morales Meseguer, Murcia, Spain); Carmen Paredes Suárez (Dermatology Department, CHUS‐Hospital Clínico Universitario de Santiago, Santiago de Compostela, Spain); María Elena Gatica Ortega (Dermatology Department, Complejo Hospitalario Universitario de Toledo, Toledo, Spain); José Juan Pereyra Rodríguez (Dermatology Department, Hospital Universitario Virgen del Rocío, Sevilla, Spain); Javier Miquel Miquel (Dermatology Department, Hospital Universitario Arnau Vilanova, Valencia, Spain); Mercedes Rodríguez Serna (Dermatology Department, Hospital Universitario La Fe, Valencia, Spain); Violeta Zaragoza Ninet (Dermatology Department Hospital General Universitario de Valencia, Valencia, Spain); Ricardo González Pérez (Dermatology Department, Hospital Universitario de Araba, Vitoria, Spain).

## References

[1] M. Bruze , L. Condé‐Salazar , A. Goossens , L. Kanerva , and I. R. White , “Thoughts on Sensitizers in a Standard Patch Test Series. The European Society of Contact Dermatitis,” Contact Dermatitis 41, no. 5 (1999): 241–250. 10554056 10.1111/j.1600-0536.1999.tb06154.x

[2] S. M. Wilkinson , S. Badulici , A. Giménez‐Arnau , et al., “The European Baseline Series: Criteria for Allergen Inclusion (With Reference to Formaldehyde Releasers),” Contact Dermatitis 85 (2021): 125–128, 10.1111/cod.13836. 33745196

[3] S. M. John , A. Bonertz , J. Zimmer , et al., “Severely Compromised Supply of Patch Test Allergens in Europe Hampers Adequate Diagnosis of Occupational and Non‐Occupational Contact Allergy. A European Society of Contact Dermatitis (ESCD), European Academy of Allergy and Clinical Immunology (EAACI), European Academy of Dermatology and Venereology (EADV) Task Forces ‘Contact Dermatitis’ and ‘Occupational Skin Disease’ Position Paper,” Contact Dermatitis 91, no. 2 (2024): 91–103, 10.1111/cod.14580. 38812248

[4] C. P. Hernández‐Fernández , P. Mercader‐García , J. F. Silvestre Salvador , et al., “Candidate Allergens for Inclusion in the Spanish Standard Series Based on Data From the Spanish Contact Dermatitis Registry,” Actas Dermo‐Sifiliográficas (English Edition) 112, no. 9 (2021): 798–805, 10.1016/j.adengl.2021.07.013. 34029518

[5] M. Wilkinson , M. Gonçalo , O. Aerts , et al., “The European Baseline Series and Recommended Additions: 2019,” Contact Dermatitis 80, no. 1 (2019): 1–4, 10.1111/cod.13155. 30421432

[6] S. M. Wilkinson , M. Gonçalo , O. Aerts , et al., “The European Baseline Series and Recommended Additions: 2023,” Contact Dermatitis 88, no. 2 (2023): 87–92, 10.1111/cod.14255. 36443008

[7] W. Uter , S. M. Wilkinson , O. Aerts , et al., “Patch Test Results With the European Baseline Series, 2019/20‐Joint European Results of the ESSCA and the EBS Working Groups of the ESCD, and the GEIDAC,” Contact Dermatitis 87, no. 4 (2022): 343–355. 35678309 10.1111/cod.14170

[8] W. Uter , J. C. Amario‐Hita , A. Balato , et al., “European Surveillance System on Contact Allergies (ESSCA): Results With the European Baseline Series, 2013/2014,” Journal of the European Academy of Dermatology and Venereology: JEADV 31, no. 9 (2017): 1516–1525, 10.1111/jdv.14423. 28627111

[9] W. Uter , A. Bauer , A. Belloni Fortina , et al., “Patch Test Results With the European Baseline Series and Additions Thereof in the ESSCA Network, 2015–2018,” Contact Dermatitis 84, no. 2 (2021): 109–120, 10.1111/cod.13704. 32945543

[10] W. Uter , S. M. Wilkinson , O. Aerts , et al., “Patch Test Results With the European Baseline Series, 2019/20‐Joint European Results of the ESSCA and the EBS Working Groups of the ESCD, and the GEIDAC,” Contact Dermatitis 87, no. 4 (2022): 343–355, 10.1111/cod.14170. 35678309

[11] W. Uter , A. Schnuch , A. Giménez‐Arnau , D. Orton , and B. Statham , “Databases and Networks: The Benefit for Research and Quality Assurance in Patch Testing,” in Contact Dermatitis, 6th ed., ed. J. D. Johansen , V. Mahler , J.‐P. Lepoittevin , and P. J. Frosch (Springer, 2020), 1–16.

[12] W. Uter , A. Schnuch , M. Wilkinson , A. Dugonik , B. Dugonik , and T. Ganslandt , “Registries in Clinical Epidemiology: The European Surveillance System on Contact Allergies (ESSCA),” Methods of Information in Medicine 55, no. 2 (2016): 193–199. 26905626 10.3414/ME15-01-0099

[13] W. Uter , “Contact Dermatitis Research Groups,” in Contact Dermatitis, 6th ed., ed. J. D. Johansen , V. Mahler , J. P. Lepoittevin , and P. J. Frosch (Springer, 2020), 1–4.

[14] J. D. Johansen , K. Aalto‐Korte , T. Agner , et al., “European Society of Contact Dermatitis Guideline for Diagnostic Patch Testing – Recommendations on Best Practice,” Contact Dermatitis 73, no. 4 (2015): 195–221. 26179009 10.1111/cod.12432

[15] W. Uter , K. E. Andersen , R. Brans , et al., “The ‘MOAHLFA(P) Index’: An Attempt to Standardise a Widely Used Array of Descriptors of Patch‐Tested Patients,” Contact Dermatitis 92, no. 4 (2025): 251–260, 10.1111/cod.14750. 39800943 PMC11880876

[16] W. Uter , R. Arnold , J. Wilkinson , et al., “A Multilingual European Patch Test Software Concept: WinAlldat/ESSCA,” Contact Dermatitis 49, no. 5 (2003): 270–271. 14996062 10.1111/j.0105-1873.2003.0225n.x

[17] F. J. Navarro‐Triviño , L. Borrego , J. F. Silvestre‐Salvador , et al., “[Translated Article] Standard and Expanded Series Patch Testing Update by the Spanish Contact Dermatitis and Skin Allergy Research Group (GEIDAC),” Actas Dermo‐Sifiliográficas 115, no. 7 (2024): T712–T721, 10.1016/j.ad.2024.05.018. 38823769

[18] W. Uter , A. Schnuch , and O. Gefeller , “Guidelines for the Descriptive Presentation and Statistical Analysis of Contact Allergy Data,” Contact Dermatitis 51, no. 2 (2004): 47–56, 10.1111/j.0105-1873.2004.00406.x. 15373843

[19] O. Gefeller , A. B. Pfahlberg , and W. Uter , “What Can Be Learnt From Nothing? – A Statistical Perspective,” Contact Dermatitis 69, no. 6 (2013): 350–354. 23848408 10.1111/cod.12112

[20] W. Uter , J. Schwitulla , J. P. Thyssen , P. J. Frosch , B. Statham , and A. Schnuch , “The ‘Overall Yield’ With the Baseline Series ‐ A Useful Addition to the Array of MOAHLFA Factors Describing Departmental Characteristics of Patch Tested Patients,” Contact Dermatitis 65, no. 6 (2011): 322–328. 22077434 10.1111/j.1600-0536.2011.01964.x

[21] A. Schnuch , J. Geier , W. Uter , et al., “National Rates and Regional Differences in Sensitization to Allergens of the Standard Series. Population‐Adjusted Frequencies of Sensitization (PAFS) in 40,000 Patients From a Multicenter Study (IVDK),” Contact Dermatitis 37, no. 5 (1997): 200–209. 9412746 10.1111/j.1600-0536.1997.tb02435.x

[22] J. García‐Gavín , J. C. Armario‐Hita , V. Fernández‐Redondo , et al., “Nickel Allergy in Spain Needs Active Intervention,” Contact Dermatitis 64, no. 5 (2011): 289–291, 10.1111/j.1600-0536.2010.01865.x. 21480915

[23] P. Basso , M. Mauro , A. Miani , A. Belloni Fortina , M. T. Corradin , and F. Larese Filon , “Sensitization to Nickel in the Triveneto Region: Temporal Trend After European Union Regulations,” Contact Dermatitis 82, no. 4 (2020): 247–250, 10.1111/cod.13450. 31811647

[24] M. G. Ahlström , M. Wennervaldt , G. McCombie , P. Blaser , and C. Lidén , “Regulatory Action Needed to Combat Nickel Contact Allergy in the Population,” Contact Dermatitis 89, no. 1 (2023): 77–78, 10.1111/cod.14330. 37137290

[25] A. B. Simonsen , J. D. Johansen , M. Deleuran , C. G. Mortz , L. Skov , and M. Sommerlund , “Children With Atopic Dermatitis May Have Unacknowledged Contact Allergies Contributing to Their Skin Symptoms,” Journal of the European Academy of Dermatology and Venereology: JEADV 32, no. 3 (2018): 428–436, 10.1111/jdv.14737. 29222945

[26] J. Geier , S. Schubert , J. Rieker‐Schwienbacher , et al., “Declining Frequency of Sensitization to Fragrance Mixes I and II: IVDK‐Data of the Years 2012–2021,” Contact Dermatitis 90, no. 5 (2024): 470–478, 10.1111/cod.14493. 38146081

[27] S. Botvid , N. H. Bennike , A. B. Simonsen , J. D. Johansen , and W. Uter , “Contact Sensitization to Fragrance Mix I and Fragrance Mix II Among European Dermatitis Patients: A Systematic Review,” Contact Dermatitis 91, no. 3 (2024): 177–185, 10.1111/cod.14618. 38945918

[28] P. J. Frosch , S. C. Rastogi , C. Pirker , et al., “Patch Testing With a New Fragrance Mix ‐ Reactivity to the Individual Constituents and Chemical Detection in Relevant Cosmetic Products,” Contact Dermatitis 52, no. 4 (2005): 216–225, 10.1111/j.0105-1873.2005.00563.x. 15859994

[29] M. G. Ahlström , W. Uter , M. G. Ahlström , and J. D. Johansen , “Decrease of Contact Allergy to Hydroxyisohexyl 3‐Cyclohexene Carboxaldehyde in Europe Prior to Its Ban and Diagnostic Value,” Contact Dermatitis 84, no. 6 (2021): 419–422, 10.1111/cod.13786. 33453125

[30] C. P. Hernández Fernández , L. Borrego , A. M. Giménez Arnau , et al., “Should Hydroxyisohexyl 3‐Cyclohexene Carboxaldehyde (Lyral) Still Be Part of the Baseline Series?,” Actas Dermo‐Sifiliográficas 116, no. 10 (2025): T1084–T1092, 10.1016/j.ad.2024.10.063. 41076186

[31] L. Stingeni , K. Hansel , M. Corazza , et al., “Contact Allergy to Hydroxyisohexyl 3‐Cyclohexene Carboxaldehyde in Italy: Prevalence, Trend, and Concordance With Fragrance Mix II,” Contact Dermatitis 88, no. 2 (2023): 129–133, 10.1111/cod.14240. 36305627

[32] I. A. Ogueta , J. Brared Christensson , E. Giménez‐Arnau , et al., “Limonene and Linalool Hydroperoxides Review: Pros and Cons for Routine Patch Testing,” Contact Dermatitis 87, no. 1 (2022): 1–12, 10.1111/cod.14064. 35122274

[33] T. Sukakul , M. Bruze , M. Mowitz , et al., “Contact Allergy to Oxidized Linalool and Oxidized Limonene: Patch Testing in Consecutive Patients With Dermatitis,” Contact Dermatitis 86, no. 1 (2022): 15–24, 10.1111/cod.13980. 34561893

[34] E. Noë , S. Huygens , M. A. Morren , M. Garmyn , A. Goossens , and L. Gilissen , “Contact Allergy in a Paediatric Population Observed in a Tertiary Referral Centre in Belgium,” Contact Dermatitis 86, no. 1 (2022): 3–8, 10.1111/cod.13975. 34537955

[35] F. Guarneri , M. Corazza , L. Stingeni , et al., “ *Myroxylon pereirae* (Balsam of Peru): Still Worth Testing?,” Contact Dermatitis 85, no. 3 (2021): 269–273, 10.1111/cod.13839. 33748955 PMC8453940

[36] SCCS (ScientificC on CS) , “SCCS (Scientific Committee on Consumer Safety), Opinion on Citral (CAS No. 5392‐40‐5, EC No. 226‐394‐6) ‐ Sensitisation Endpoint, Preliminary Version of 27 March 2024, Final Version of 29 July 2024, SCCS/1666/24.” 2024.

[37] W. Uter , A. C. Figueiredo , A. Belloni Fortina , et al., “Extended Fragrance Ingredients Surveillance Study (EFISS)‐Protocol for a Clinical Surveillance Study on Contact Allergy to 7 Fragrance Materials in Widespread Use but Hitherto Not Systematically Patch Tested,” Archives of Dermatological Research 317, no. 1 (2025): 778, 10.1007/s00403-025-04286-9. 40407895 PMC12102106

[38] A. Goossens and O. Aerts , “Contact Allergy to and Allergic Contact Dermatitis From Formaldehyde and Formaldehyde Releasers: A Clinical Review and Update,” Contact Dermatitis 87, no. 1 (2022): 20–27, 10.1111/cod.14089. 35229319

[39] R. Søgaard , P. B. Poulsen , R. M. Gelardi , S. Geschke , J. F. B. Schwensen , and J. D. Johansen , “Hidden Formaldehyde in Cosmetic Products,” Contact Dermatitis 91, no. 6 (2024): 497–502, 10.1111/cod.14669. 39155513

[40] M. E. Gatica‐Ortega , M. A. Pastor‐Nieto , P. Beneyto , and L. Borrego , “Allergic Contact Dermatitis to Incontinence Pads in a Patient Sensitized to Multiple (Meth)acrylates and Formaldehyde,” Contact Dermatitis 88, no. 5 (2023): 413–415, 10.1111/cod.14288. 36727702

[41] I. Hauksson , A. Pontén , B. Gruvberger , M. Isaksson , and M. Bruze , “Clinically Relevant Contact Allergy to Formaldehyde May Be Missed by Testing With Formaldehyde 1·0%,” British Journal of Dermatology 164, no. 3 (2011): 568–572, 10.1111/j.1365-2133.2010.10151.x. 21114477

[42] M. Frick‐Engfeldt , B. Gruvberger , M. Isaksson , I. Hauksson , A. Pontén , and M. Bruze , “Comparison of Three Different Techniques for Application of Water Solutions to Finn Chambers,” Contact Dermatitis 63, no. 5 (2010): 284–288, 10.1111/j.1600-0536.2010.01797.x. 20946457

[43] M. D. Lynch , J. P. McFadden , J. M. White , P. Banerjee , and I. R. White , “Age‐Specific Profiling of Cutaneous Allergy at High Temporal Resolution Suggests Age‐Related Alterations in Regulatory Immune Function,” Journal of Allergy and Clinical Immunology 140, no. 5 (2017): 1451–1453, 10.1016/j.jaci.2017.03.054. 28606588

[44] C. Lidén and I. R. White , “Comment on MDBGN/DBDCB, the European Baseline Series, and EU Legislation,” Contact Dermatitis 85, no. 5 (2021): 607–610, 10.1111/cod.13937. 34250623

[45] A. M. Giménez‐Arnau , G. Deza , A. Bauer , et al., “Contact Allergy to Preservatives: ESSCA* Results With the Baseline Series, 2009–2012,” Journal of the European Academy of Dermatology and Venereology: JEADV 31, no. 4 (2017): 664–671, 10.1111/jdv.14063. 27896884

[46] M. J. Reeder , E. Warshaw , S. Aravamuthan , et al., “Trends in the Prevalence of Methylchloroisothiazolinone/Methylisothiazolinone Contact Allergy in North America and Europe,” JAMA Dermatology 159, no. 3 (2023): 267–274, 10.1001/jamadermatol.2022.5991. 36652228 PMC9857829

[47] C. Lidén and I. R. White , “Increasing Non‐Cosmetic Exposure and Sensitization to Isothiazolinones Require Action for Prevention: Review,” Contact Dermatitis 90, no. 5 (2024): 445–457, 10.1111/cod.14523. 38382085

[48] M. Edlund , M. Holm , A. Inerot , L. Långsved , A. Dahlman‐Höglund , and L. Hagvall , “Contact Sensitization and Self‐Reported Eczema in Swedish Painters With Occupational Exposure to Isothiazolinones,” Contact Dermatitis 91, no. 2 (2024): 126–132, 10.1111/cod.14572. 38769738

[49] L. F. Soriano , S. K. Soriano , and D. A. Buckley , “Ironing Water: An Under‐Recognized Source of Contact Allergens,” Contact Dermatitis 88, no. 1 (2023): 75–76, 10.1111/cod.14225. 36124850

[50] R. André , Y. Alipour Tehrany , A. Bugey , P. Edder , and P. Piletta , “Hand Dermatitis Aggravated by Contact Allergy to Methylisothiazolinone in Protective Nitrile Gloves,” Contact Dermatitis 87, no. 4 (2022): 383–384, 10.1111/cod.14173. 35704221 PMC9546190

[51] L. F. Soriano , S. K. Soriano , and D. A. Buckley , “Dog Cosmetics: Another Unexpected Source of Allergen Exposure,” Contact Dermatitis 88, no. 6 (2023): 496–499, 10.1111/cod.14307. 37015256

[52] C. Foti , W. A. Rosato , G. Sbarra , P. Romita , and A. Antelmi , “A Case of Allergic Contact Dermatitis to Isothiazolinones Contained in a Haemorrhoid Cream,” Contact Dermatitis 90, no. 6 (2024): 613–615, 10.1111/cod.14519. 38332250

[53] G. Fenech , L. Bensefa‐Colas , and M. N. Crepy , “Allergic Contact Dermatitis Caused by Isothiazolinones in an Ultrasound Gel: Need for Stricter Regulation of Topical Medical Devices,” Contact Dermatitis 92, no. 6 (2025): 483–485, 10.1111/cod.14771. 39935102 PMC12055312

[54] I. G. Nunes , M. Relvas , and M. Gonçalo , “Should Paraben Mix be Removed From the European Baseline Series,” Acta Dermatovenerologica Croatica: ADC 29, no. 3 (2021): 171–172. 34990349

[55] C. Kursawe Larsen , J. F. B. Schwensen , C. Zachariae , and J. D. Johansen , “Contact Allergy to Rubber Accelerators in Consecutively Patch Tested Danish Eczema Patients: A Retrospective Observational Study From 1990 to 2019,” Contact Dermatitis 90, no. 2 (2024): 116–125, 10.1111/cod.14421. 37735996

[56] C. Kursawe Larsen , J. F. B. Schwensen , C. Zachariae , C. Svedman , J. D. Johansen , and O. Bergendorff , “Contents of Sensitising Rubber Accelerators in Disposable Rubber Gloves: A Copenhagen Market Survey,” Contact Dermatitis 92, no. 2 (2025): 131–136, 10.1111/cod.14709. 39367740 PMC11710924

[57] C. Kursawe Larsen , A. B. Funch , H. Vaher , et al., “Cross‐Reactivity Between Thiuram Disulfides and Dithiocarbamates. A Study of TETD and ZDEC Using Mouse Models,” Contact Dermatitis 92, no. 2 (2025): 137–144, 10.1111/cod.14706. 39340203 PMC11710922

[58] K. L. Warburton , W. Uter , J. Geier , et al., “Patch Testing With Rubber Series in Europe: A Critical Review and Recommendation,” Contact Dermatitis 76, no. 4 (2017): 195–203, 10.1111/cod.12736. 28032352

[59] W. Uter , K. Warburton , E. Weisshaar , et al., “Patch Test Results With Rubber Series in the European Surveillance System on Contact Allergies (ESSCA), 2013/2014,” Contact Dermatitis 75, no. 6 (2016): 345–352, 10.1111/cod.12651. 27402420

[60] Anonymous , “Mercapto Mix,” Contact Dermatitis 32 (1995): 255.

[61] D. Pesqué , N. Planella‐Fontanillas , L. Borrego , et al., “Patch Test Results to the Spanish Baseline Patch Test Series According to Age Groups: A Multicentric Prospective Study From 2019 to 2023,” Contact Dermatitis 92, no. 2 (2025): 120–130, 10.1111/cod.14702. 39394969 PMC11710926

[62] W. Boonchai , S. Likittanasombat , N. Viriyaskultorn , and S. Kanokrungsee , “Gender Differences in Allergic Contact Dermatitis to Common Allergens,” Contact Dermatitis 90, no. 5 (2024): 458–465, 10.1111/cod.14479. 38109794

[63] A. T. Karlberg , M. H. Albadr , and U. Nilsson , “Tracing Colophonium in Consumer Products,” Contact Dermatitis 85, no. 6 (2021): 671–678, 10.1111/cod.13944. 34291483

[64] S. Traidl , T. Werfel , F. Ruëff , et al., “Patch Test Results in Patients With Suspected Contact Allergy to Shoes: Retrospective IVDK Data Analysis 2009–2018,” Contact Dermatitis 85, no. 3 (2021): 297–306, 10.1111/cod.13868. 33882155

[65] I. Hamid and N. Stone , “Bees Wax Wraps‐A Novel Source of Colophonium Allergic Contact Dermatitis,” Contact Dermatitis 88, no. 4 (2023): 315–316, 10.1111/cod.14267. 36524719

[66] T. B. J. Eriksson , M. Isaksson , M. Engfeldt , et al., “Contact Allergy in Swedish Professional Ice Hockey Players,” Contact Dermatitis 90, no. 6 (2024): 574–584, 10.1111/cod.14529. 38501375

[67] C. Svedman , J. Dahlin , N. Hamnerius , U. N. A. R. Adwa , and M. Bruze , “Occupational Allergic Contact Dermatitis Induced by Adhesives Used for Prevention of Chafing in the Military Forces‐A Case Report,” Contact Dermatitis 89, no. 5 (2023): 391–393, 10.1111/cod.14398. 37574207

[68] E. Dendooven , S. Kerre , A. Goossens , and O. Aerts , “Allergic Contact Dermatitis From a Medical Device Containing *Picea abies* (Norway Spruce) Resin: Skin Sensitizers Other Than Resin Acids Might Be of Importance,” Contact Dermatitis 88, no. 1 (2023): 60–62, 10.1111/cod.14215. 36068985

[69] C. Huang and J. DeKoven , “An Unexpected Source of Glucose Monitor‐Associated Allergic Contact Dermatitis,” Contact Dermatitis 85 (2021): 235–236, 10.1111/cod.13815. 33598976

[70] O. Aerts , E. Dendooven , and N. Raison‐Peyron , “Sensitization to Modified Colophonium in Glucose Sensors: Another Problem for Diabetes Patients,” Contact Dermatitis 87, no. 6 (2022): 553–555, 10.1111/cod.14213. 36068926

[71] A. Bauer , M. Pesonen , R. Brans , et al., “Occupational Contact Allergy: The European Perspective‐Analysis of Patch Test Data From ESSCA Between 2011 and 2020,” Contact Dermatitis 88, no. 4 (2023): 263–274, 10.1111/cod.14280. 36694979

[72] K. E. Dear , C. Felmingham , C. Ronaldson , and R. L. Nixon , “Presentations to Emergency Departments in Melbourne, Australia Diagnosed as Allergic Contact Dermatitis,” Contact Dermatitis 88, no. 2 (2023): 145–149, 10.1111/cod.14230. 36193797

[73] A. G. Christiansen , M. B. Kinnerup , O. Carstensen , et al., “Occupational Exposure to Epoxy Components and Risk of Dermatitis: A Registry‐Based Follow‐Up Study of the Wind Turbine Industry,” Contact Dermatitis 90, no. 1 (2024): 32–40, 10.1111/cod.14431. 37795841

[74] S. Schubert , A. Bauer , U. Hillen , et al., “Occupational Contact Dermatitis in Painters and Varnishers: Data From the Information Network of Departments of Dermatology (IVDK), 2000 to 2019,” Contact Dermatitis 85, no. 5 (2021): 494–502, 10.1111/cod.13935. 34260080

[75] I. Temam , O. Bauvin , and C. Boulard , “Epoxy Resin, an Emerging Allergen in Women?,” Contact Dermatitis 89, no. 6 (2023): 503–505, 10.1111/cod.14410. 37611919

[76] Q. Samaran , R. Strizzolo , O. Dereure , and N. Raison‐Peyron , “Non‐Occupational Allergic Contact Dermatitis to Epoxy Resin in a Cosplayer,” Contact Dermatitis 89, no. 2 (2023): 122–124, 10.1111/cod.14329. 37121570

[77] M. Coco‐Viloin , M. Severino‐Freire , and F. Giordano‐Labadie , “Non‐Occupational Allergic Contact Dermatitis From Epoxy Resin in Children's Games,” Contact Dermatitis 88, no. 3 (2023): 232–234, 10.1111/cod.14254. 36444522 PMC10108247

[78] A. C. de Groot and T. Rustemeyer , “2‐Hydroxyethyl Methacrylate (HEMA): A Clinical Review of Contact Allergy and Allergic Contact Dermatitis. Part 2. Cross‐ and Co‐Sensitization, Other Skin Reactions to HEMA, Position of HEMA Among (Meth)acrylates, Sensitivity as Screening Agent, Presence of HEMA in Commercial Products and Practical Information on Patch Test Procedures,” Contact Dermatitis 90, no. 1 (2024): 1–16, 10.1111/cod.14430. 37778325

[79] S. M. Wilkinson , O. Aerts , T. Agner , et al., “Contact Allergy to Methacrylate Containing Nail Products: Lack of Impact of EU Legislation. An Audit on Behalf of the European Environmental Contact Dermatitis Research Group (EECDRG),” Contact Dermatitis 92, no. 4 (2025): 283–290, 10.1111/cod.14745. 39721608

[80] I. M. Steunebrink , A. de Groot , and T. Rustemeyer , “Presence of 2‐Hydroxyethyl Methacrylate (HEMA) and Other (Meth)acrylates in Nail Cosmetics, and Compliance With EU Legislation: An Online Market Survey,” Contact Dermatitis 90, no. 1 (2024): 60–65, 10.1111/cod.14441. 37848187

[81] A. C. de Groot and T. Rustemeyer , “2‐Hydroxyethyl Methacrylate (HEMA): A Clinical Review of Contact Allergy and Allergic Contact Dermatitis‐Part 1. Introduction, Epidemiology, Case Series and Case Reports,” Contact Dermatitis 89, no. 6 (2023): 401–433, 10.1111/cod.14405. 37752620

[82] W. Uter , M. Worm , R. Brans , et al., “Patch Test Results With Caine Mix III and Its Three Constituents in Consecutive Patients of the IVDK,” Contact Dermatitis 84, no. 6 (2021): 481–483, 10.1111/cod.13778. 33400817

[83] P. Mercader‐García , M. A. Pastor‐Nieto , I. García‐Doval , et al., “Are the Spanish Baseline Series Markers Sufficient to Detect Contact Allergy to Corticosteroids in Spain? A GEIDAC Prospective Study,” Contact Dermatitis 78, no. 1 (2018): 76–82, 10.1111/cod.12874. 28960334

[84] P. Mercader‐García , J. F. Silvestre , F. J. Navarro‐Triviño , et al., “A Re‐Assessment of the Value of Markers of Corticosteroid Contact Allergy in the Spanish Baseline Series: Clobetasol Propionate in the Spotlight,” Contact Dermatitis 91, no. 3 (2024): 228–236, 10.1111/cod.14639. 38965446

[85] W. Uter , R. Spiewak , S. M. Cooper , et al., “Contact Allergy to Ingredients of Topical Medications: Results of the European Surveillance System on Contact Allergies (ESSCA), 2009–2012,” Pharmacoepidemiology and Drug Safety 25, no. 11 (2016): 1305–1312, 10.1002/pds.4064. 27464585

[86] K. Trimeche , I. Lahouel , H. Belhadjali , N. B. Salah , M. Youssef , and J. Zili , “Contact Allergy in Atopic Dermatitis: A Prospective Study on Prevalence, Incriminated Allergens and Clinical Insights,” Contact Dermatitis 90, no. 5 (2024): 514–519, 10.1111/cod.14494. 38151921

[87] J. Ruiz Sánchez , J. Espiñeira Sicre , V. Cristóbal Redondo , I. Albert Cobo , O. Al‐Wattar Ceballos , and J. F. Silvestre Salvador , “Chronic Prurigo Associated With Allergic Contact Dermatitis: A Case Series Highlighting Textile Dyes and Fragrance Allergens,” Contact Dermatitis 92, no. 6 (2025): 469–474, 10.1111/cod.14764. 39916502

[88] M. Isaksson , A. Antelmi , J. Dahlin , et al., “Exclusion of Disperse Orange 3 Is Possible From the Textile Dye Mix Present in the Swedish Baseline Patch Test Series. A Study by the Swedish Contact Dermatitis Research Group,” Contact Dermatitis 88, no. 1 (2023): 54–59, 10.1111/cod.14223. 36112512 PMC10091765

[89] G. S. A. Nyman , A. M. Giménez‐Arnau , J. Grigaitiene , L. Malinauskiene , E. Paulsen , and L. Hagvall , “Patch Testing With Propolis of Different Geographical Origins in a Baseline Series,” Acta Dermato‐Venereologica 101, no. 11 (2021): adv00591, 10.2340/actadv.v101.423. 34664078 PMC9455323

[90] S. Schubert , J. Geier , H. Dickel , T. Buhl , F. Ruëff , and H. Löffler , “Contact Sensitization to Propolis in the Information Network of Departments of Dermatology (IVDK) 2013 to 2019 and Market Survey of Propolis Commerce in Germany,” Contact Dermatitis 85, no. 6 (2021): 722–724, 10.1111/cod.13960. 34423862

[91] K. Piontek , S. Radonjic‐Hoesli , J. Grabbe , et al., “Comparison of Patch Testing Brazilian (Green) Propolis and Chinese (Poplar‐Type) Propolis: Clinical Epidemiological Study Using Data From the Information Network of Departments of Dermatology (IVDK),” Contact Dermatitis 92, no. 3 (2025): 209–216, 10.1111/cod.14701. 39367763

[92] E. Paulsen and K. E. Andersen , “Screening for Compositae Contact Sensitization With Sesquiterpene Lactones and Compositae Mix 2.5% Pet,” Contact Dermatitis 81, no. 5 (2019): 368–373, 10.1111/cod.13346. 31265134

[93] E. Paulsen , “The Sesquiterpene Lactone Mix: A Review of Past, Present and Future Aspects,” Contact Dermatitis 89, no. 6 (2023): 434–441, 10.1111/cod.14419. 37820718

[94] E. Paulsen and C. G. Mortz , “The Value of Patch Testing With Plants “as is” in Diagnosing Plant Sensitization,” Contact Dermatitis 91, no. 6 (2024): 459–464, 10.1111/cod.14680. 39294867

[95] E. Dendooven , E. Dirinck , K. Foubert , and O. Aerts , ““Re‐Testing” Suggests That Cosensitizations to Isobornyl Acrylate and Sesquiterpene Lactones May Be due to Cross‐Reactivity,” Contact Dermatitis 86, no. 1 (2022): 57–59, 10.1111/cod.13972. 34519361

[96] C. C. A. van Amerongen , R. Ofenloch , D. Dittmar , and M. L. A. Schuttelaar , “New Positive Patch Test Reactions on Day 7‐the Additional Value of the Day 7 Patch Test Reading,” Contact Dermatitis 81, no. 4 (2019): 280–287, 10.1111/cod.13322. 31116435 PMC6771944

[97] S. Forkel , S. Schubert , H. Dickel , et al., “The Benefit of Late Readings in Patch Testing Depends Both on Allergen and Patient Characteristics,” Allergy 77, no. 5 (2021): 1477–1485, 10.1111/all.15149. 34687560

[98] W. Uter , “Fragrance Mix I: TRUE Test() Versus Pet.‐Based Patch Test,” Contact Dermatitis 72, no. 4 (2015): 256–258, 10.1111/cod.12352. 25676415

[99] D. Dittmar and M. L. Schuttelaar , “Comparing Patch Test Results of Methylchloroisothiazolinone/Methylisothiazolinone Tested With Both TRUE Test and 100 Ppm Using Investigator‐Loaded Chambers,” Contact Dermatitis 78, no. 2 (2018): 159–161, 10.1111/cod.12871. 29341185

[100] A. F. Fransway , K. A. Zug , D. V. Belsito , et al., “North American Contact Dermatitis Group Patch Test Results for 2007–2008,” Dermatitis: Contact, Atopic, Occupational, Drug 24, no. 1 (2013): 10–21, 10.1097/DER.0b013e318277ca50. 23340394

[101] R. Spiewak , “Patch Testing for Contact Allergy and Allergic Contact Dermatitis,” Open Allergy Journal 1, no. 1 (2008): 42–51.

[102] W. Uter , A. Bauer , L. Bensefa‐Colas , et al., “Pilot Study on a New Concept of Documenting the Clinical Relevance of Patch Test Results in Contact Dermatitis Patients,” Contact Dermatitis 79, no. 6 (2018): 370–377, 10.1111/cod.13097. 30141249

