# Supplementary material for: Patch Test Results With the European Baseline Series, 2021/2022—Joint European Results of the ESSCAA and the EBSB Working Groups of the ESCD, and the GEIDACC
Source: Contact Dermatitis. 2026 Mar 12;95(1):17–32. doi: 10.1111/cod.70134 (PMC13238334; doi:10.1111/cod.70134)
Supplement: Supplementary file 1 — Table S1: Contribution by department and country, respectively. Additional contributions are from the “Grupo Español de Investigación en Dermatitis de Contacto y Alergia Cutánea” (GEIDAC):6 (i) additional 2865 patients tested with investigator‐loaded system, and (ii) 2702 using the TRUE Test in 2021/22. Table S2: Patch test results (day 3 to day 5) with the European Baseline series, 2021–2022, stratified for country. Conc., concentration in %, all tested in pet., except where indicated otherwise: a, aqua. [file COD-95-17-s001.docx]

**Online supplemental Table S1**: Contribution by department and country, respectively. Additional contributions are from the “Grupo Español de Investigación en Dermatitis de Contacto y Alergia Cutánea” (GEIDAC):^6^ (i) additional 2865 patients tested with investigator-loaded system, and (ii) 2702 using the TRUE Test in 2021/22.

| Country | City | 2021 tested | 2022 tested |
| --- | --- | --- | --- |
| Austria | Graz | 98 | 90 |
| Belgium | Antwerp | 400 | 491 |
| Switzerland | Basel | 148 | 132 |
| Switzerland | Bern | 314 | 322 |
| Switzerland | Aarau | 117 | 104 |
| Switzerland | Lausanne/Epalignes | 40 | 25 |
| Germany | Dortmund | 64 | 37 |
| Germany | Göttingen | 83 | 89 |
| Germany | Kiel | 191 | 79 |
| Germany | Dresden | 400 | 360 |
| Germany | Jena | 149 | 150 |
| Germany | Mainz | 118 | 121 |
| Germany | Osnabrück | 352 | 318 |
| Germany | Erlangen | 254 | 239 |
| Germany | Heidelberg | 79 | 66 |
| Germany | Hannover | 162 | 138 |
| Germany | Bochum | 305 | 361 |
| Greece | Athens |  | 346 |
| Greece | Heraklion, Crete | 54 | 101 |
| Hungary | Budapest | 247 | 262 |
| Italy | Napoli | 415 | 430 |
| Italy | Padova Paediatrica | 43 | 44 |
| Italy | Trieste | 258 | 307 |
| Italy | Genova | 350 | 421 |
| Italy | Perugia | 382 | 180 |
| Lithuania | Kaunas | 310 | 391 |
| The Netherlands | Groningen | 274 | 264 |
| The Netherlands | Amsterdam VU | 905 | 844 |
| Poland | Krakow | 41 | 44 |
| Portugal | Coimbra | 273 | 253 |
| Slovenia | Maribor/Univ. | 140 | 243 |
| Slovenia | Maribor/Clinic | 110 | 77 |
| Slovenia | Celje | 374 | 421 |
| Slovenia | Novo Mesto | 118 |  |
| Slovenia | Ljubljana/Clinic | 10 |  |
| United Kingdom | Leicester | 83 | 180 |
| United Kingdom | Middlesbrough | 193 |  |
| United Kingdom | Leeds | 869 | 1008 |

**Online supplemental Table S2**: Patch test results (day 3 to day 5) with the European Baseline series, 2021–2022, stratified for country. Conc., concentration in %, all tested in pet., except where indicated otherwise: ^a^, aqua

| **Allergen** | **Conc** | **AT tested** | **AT % pos. (95% CI)** | **BE tested** | **BE % pos. (95% CI)** | **CH tested** | **CH % pos. (95% CI)** | **DE tested** | **DE % pos. (95% CI)** |
| --- | --- | --- | --- | --- | --- | --- | --- | --- | --- |
| **Metals** |  |  |  |  |  |  |  |  |  |
| Potassium dichromate | 0.5 | 182 | 7.14 (3.86-11.9) | 891 | 4.83 (3.51-6.45) | 1071 | 5.04 (3.81-6.53) | 3239 | 5.37 (4.62-6.21) |
| Cobalt chloride | 1.0 | 182 | 15.38 (10.47-21.46) | 891 | 6.96 (5.38-8.83) | 1094 | 8.14 (6.58-9.92) | 3255 | 7.99 (7.08-8.97) |
| Nickel sulphate | 5.0 | 182 | 25.3 (19.1-32.2) | 891 | 18.5 (16.0-21.2) | 1108 | 21.8 (19.4-24.3) | 3389 | 15.9 (14.7-17.2) |
| **Fragrances** |  |  |  |  |  |  |  |  |  |
| Fragrance mix I | 8.0 | 183 | 8.74 (5.08-13.8) | 891 | 12.1 (10.1-14.5) | 1109 | 9.20 (7.56-11.1) | 3221 | 4.94 (4.21-5.74) |
| Fragrance mix II | 14 | 183 | 4.92 (2.27-9.13) | 891 | 5.05 (3.71-6.70) | 1018 | 3.34 (2.32-4.64) | 3382 | 3.19 (2.63-3.84) |
| HICC | 5.0 | 182 | 1.65 (0.34-4.74) | 891 | 1.57 (0.86-2.62) | 990 | 0.71 (0.28-1.45) | 3273 | 1.34 (0.98-1.8) |
| *Myroxylon pereirae* (balsam of Peru) | 25 | 182 | 9.34 (5.54-14.53) | 891 | 14.7 (12.44-17.2) | 1093 | 10.34 (8.6-12.3) | 3416 | 6.50 (5.70-7.38) |
| **Preservatives** |  |  |  |  |  |  |  |  |  |
| Formaldehyde | 1.0^a^ | 182 | 0.55 (0.01-3.02) | 0 |  | 1093 | 1.01 (0.50-1.79) | 3405 | 0.94 (0.64-1.32) |
| Formaldehyde | 2.0^a^ | 0 |  | 891 | 3.7 (2.56-5.16) | 0 |  | 0 |  |
| MCI/MI 3:1 | 0.01^a^ | 183 | 4.37 (1.91-8.43) | 891 | 6.51 (4.98-8.33) | 1108 | 2.26 (1.47-3.31) | 3296 | 2.43 (1.93-3.01) |
| MCI/MI 3:1 | 0.02^a^ | 0 |  | 0 |  | 0 |  | 0 |  |
| Methylisothiazolinone | 0.05^a^ | 183 | 4.92 (2.27-9.13) | 0 |  | 1110 | 3.33 (2.36-4.57) | 3069 | 3.42 (2.81-4.13) |
| Methylisothiazolinone | 0.20^a^ | 0 |  | 891 | 5.84 (4.39-7.58) | 0 |  | 0 |  |
| Paraben mix | 16 | 0 |  | 891 | 0.22 (0.03-0.81) | 0 |  | 0 |  |
| Quaternium-15 | 1.0 | 0 |  | 891 | 0.34 (0.07-0.98) | 0 |  | 0 |  |
| Methyldibromo glutaronitrile | 0.3 | 0 |  | 0 |  | 0 |  | 0 |  |
| Methyldibromo glutaronitrile | 0.5 | 0 |  | 895 | 7.93 (6.25-9.9) | 0 |  | 0 |  |
| **Medicaments, excipients** |  |  |  |  |  |  |  |  |  |
| Caine mix III (Benzo-,Cincho-,Tetracaine) | 10 | 0 |  | 891 | 0.45 (0.12-1.15) | 0 |  | 0 |  |
| Budesonide | 0.01 | 0 |  | 891 | 0.45 (0.12-1.15) | 0 |  | 0 |  |
| Budesonide | 0.1 | 0 |  | 0 |  | 0 |  | 0 |  |
| Tixocortol pivalate | 0.1 | 0 |  | 891 | 0.56 (0.18-1.3) | 0 |  | 0 |  |
| Tixocortol pivalate | 1.0 | 0 |  | 0 |  | 0 |  | 0 |  |
| Neomycin sulphate | 20 | 0 |  | 891 | 0.34 (0.07-0.98) | 0 |  | 0 |  |
| Lanolin (wool alcohols) | 30 | 182 | 1.10 (0.13-3.91) | 891 | 1.80 (1.03-2.9) | 1066 | 1.69 (1-2.66) | 3405 | 1.32 (0.97-1.76) |
| **Rubber additives** |  |  |  |  |  |  |  |  |  |
| Thiuram mix | 1.0 | 183 | 1.64 (0.34-4.72) | 891 | 2.58 (1.64-3.85) | 1104 | 2.36 (1.54-3.43) | 3425 | 3.53 (2.94-4.21) |
| *N*-Isopropyl-*N*’-phenyl-*p*-phenylenediamine | 0.1 | 182 | 0.55 (0.01-3.02) | 891 | 0.56 (0.18-1.3) | 1092 | 0.37 (0.10-0.94) | 3274 | 0.55 (0.33-0.87) |
| Mercapto mix^(i)^ | 1.0 | 183 | 1.09 (0.13-3.89) | 0 |  | 1105 | 0.45 (0.15-1.05) | 2653 | 0.53 (0.29-0.88) |
| Mercapto mix^(ii)^ | 2.0 | 0 |  | 891 | 0.56 (0.18-1.3) | 0 |  | 0 |  |
| Mercaptobenzothiazole | 2.0 | 183 | 1.64 (0.34-4.72) | 891 | 0.45 (0.12-1.15) | 1107 | 0.45 (0.15-1.05) | 3439 | 0.79 (0.52-1.14) |
| **Resins/glues** |  |  |  |  |  |  |  |  |  |
| Colophonium | 20 | 183 | 7.10 (3.84-11.8) | 891 | 5.05 (3.71-6.7) | 1028 | 4.18 (3.04-5.59) | 3365 | 3.77 (3.16-4.47) |
| Epoxy resin | 1.0 | 182 | 2.2 (0.60-5.53) | 891 | 1.91 (1.12-3.04) | 996 | 1.91 (1.15-2.96) | 3331 | 1.83 (1.40-2.35) |
| *p*-*tert*-Butylphenol formaldehyde resin | 1.0 | 0 |  | 891 | 0.67 (0.25-1.46) | 0 |  | 0 |  |
| 2-Hydroxyethyl methacrylate | 2.0 | 0 |  | 891 | 2.92 (1.91-4.25) | 0 |  | 0 |  |
| **Other** |  |  |  |  |  |  |  |  |  |
| *p*-Phenylenediamine | 1.0 | 0 |  | 891 | 4.94 (3.61-6.57) | 0 |  | 0 |  |
| Sesquiterpene lactone mix | 0.1 | 0 |  | 891 | 0.67 (0.25-1.46) | 0 |  | 0 |  |
| Propolis | 10 | 183 | 9.29 (5.51-14.5) | 891 | 4.15 (2.94-5.68) | 1102 | 18.8 (16.5-21.2) | 3149 | 2.48 (1.96-3.08) |
| Textile dye mix | 6.6 | 0 |  | 891 | 4.71 (3.42-6.32) | 0 |  | 0 |  |

AT, Austria; BE, Belgium; CH, Switzerland; DE, Germany; epoxy resin, diglycidyl ether of bisphenol A; HICC, hydroxyisohexyl 3-cyclohexene carboxaldehyde; mercapto mix^(i)^, containing N-cyclohexylbenzothiazyl sulfenamide, dibenzothiazyl disulfide, and morpholinylmercaptobenzothiazole; mercapto mix^(ii)^, containing N-cyclohexylbenzothiazyl sulfenamide, mercaptobenzothiazole, dibenzothiazyl disulfide, and morpholinylmercaptobenzothiazole; MCI, methylchloroisothiazolinone; MI, methylisothiazolinone.

| **Allergen** | **Conc.** | **ES tested** | **ES % pos. (95% CI)** | **GR tested** | **GR % pos. (95% CI)** | **HU tested** | **HU % pos. (95% CI)** | **IT tested** | **IT % pos. (95% CI)** |
| --- | --- | --- | --- | --- | --- | --- | --- | --- | --- |
| **Metals** |  |  |  |  |  |  |  |  |  |
| Potassium dichromate | 0.5 | 2606 | 3.15 (2.51-3.89) | 501 | 4.19 (2.61-6.34) | 509 | 2.75 (1.51-4.57) | 1996 | 2.40 (1.78-3.18) |
| Cobalt chloride | 1.0 | 2656 | 5.95 (5.08-6.92) | 501 | 7.39 (5.25-10.04) | 509 | 1.77 (0.81-3.33) | 1996 | 7.31 (6.21-8.55) |
| Nickel sulphate | 5.0 | 2683 | 20.2 (18.7-21.8) | 501 | 26.4 (22.5-30.4) | 509 | 17.1 (13.9-20.7) | 1996 | 24.3 (22.4-26.2) |
| **Fragrances** |  |  |  |  |  |  |  |  |  |
| Fragrance mix I | 8.0 | 2526 | 4.16 (3.41-5.01) | 501 | 16.8 (13.6-20.3) | 509 | 4.91 (3.20-7.17) | 1996 | 3.86 (3.06-4.80) |
| Fragrance mix II | 14 | 4780 | 3.41 (2.91-3.96) | 501 | 3.59 (2.14-5.62) | 509 | 6.29 (4.34-8.76) | 1994 | 2.51 (1.87-3.29) |
| HICC | 5.0 | 4427 | 0.79 (0.55-1.10) | 155 | 0.65 (0.02-3.54) | 509 | 1.18 (0.43-2.55) | 721 | 0.83 (0.31-1.80) |
| *Myroxylon pereirae* (balsam of Peru) | 25 | 2630 | 4.75 (3.97-5.64) | 501 | 11.0 (8.38-14.1) | 509 | 4.32 (2.73-6.47) | 1997 | 3.05 (2.34-3.91) |
| **Preservatives** |  |  |  |  |  |  |  |  |  |
| Formaldehyde | 1.0^a^ | 0 |  | 0 |  | 0 |  | 565 | 1.06 (0.39-2.3) |
| Formaldehyde | 2.0^a^ | 4770 | 2.43 (2.01-2.91) | 501 | 3.59 (2.14-5.62) | 509 | 1.18 (0.43-2.55) | 1431 | 2.24 (1.53-3.14) |
| MCI/MI 3:1 | 0.01^a^ | 0 |  | 501 | 3.39 (1.99-5.38) | 509 | 4.72 (3.04-6.93) | 652 | 5.52 (3.9-7.56) |
| MCI/MI 3:1 | 0.02^a^ | 4494 | 4.25 (3.68-4.88) | 0 |  | 0 |  | 1345 | 6.02 (4.81-7.43) |
| Methylisothiazolinone | 0.05^a^ | 0 |  | 0 |  | 0 |  | 0 |  |
| Methylisothiazolinone | 0.20^a^ | 4772 | 5.95 (5.3-6.66) | 155 | 0 (0-2.35) | 509 | 0 (0-0.72) | 1431 | 2.38 (1.65-3.3) |
| Paraben mix | 16 | 0 |  | 501 | 1.60 (0.69-3.12) | 509 | 0.98 (0.32-2.28) | 1826 | 0.33 (0.12-0.71) |
| Quaternium-15 | 1.0 | 2865 | 0.42 (0.22-0.73) | 0 |  | 0 |  | 652 | 0.31 (0.04-1.10) |
| Methyldibromo glutaronitrile | 0.3 | 0 |  | 346 | 1.16 (0.32-2.93) | 509 | 1.57 (0.68-3.07) | 87 | 0 (0-4.15) |
| Methyldibromo glutaronitrile | 0.5 | 3414 | 3.05 (2.5-3.68) | 501 | 12.6 (9.80-15.8) | 0 |  | 0 |  |
| **Medicaments, excipients** |  |  |  |  |  |  |  |  |  |
| Caine mix III (Benzo-,Cincho-,Tetracaine) | 10 | 954 | 1.05 (0.50-1.92) | 501 | 1.60 (0.69-3.12) | 0 |  | 0 |  |
| Budesonide | 0.01 | 2563 | 0.70 (0.42-1.11) | 501 | 1.80 (0.82-3.38) | 509 | 0.79 (0.21-2.00) | 1431 | 0.42 (0.15-0.91) |
| Budesonide | 0.1 | 0 |  | 0 |  | 0 |  | 0 |  |
| Tixocortol pivalate | 0.1 | 2563 | 0.31 (0.13-0.61) | 155 | 0.65 (0.02-3.54) | 509 | 0.79 (0.21-2.00) | 0 |  |
| Tixocortol pivalate | 1.0 | 0 |  | 0 |  | 0 |  | 0 |  |
| Neomycin sulphate | 20 | 2524 | 0.79 (0.48-1.22) | 501 | 2.79 (1.54-4.64) | 509 | 0.20 (0-1.09) | 1997 | 0.65 (0.35-1.11) |
| Lanolin (wool alcohols) | 30 | 2524 | 0.67  (0.39-1.08) | 501 | 0.60 (0.12-1.74) | 509 | 8.64  (6.35-11.4) | 1996 | 0.65 (0.35-1.11) |
| **Rubber additives** |  |  |  |  |  |  |  |  |  |
| Thiuram mix | 1.0 | 2539 | 1.77 (1.30-2.36) | 501 | 2.99 (1.69-4.89) | 511 | 14.7 (11.7-18.1) | 1996 | 2.10 (1.52-2.83) |
| *N*-Isopropyl-*N*’-phenyl-*p*-phenylenediamine | 0.1 | 2642 | 0.79 (0.49-1.21) | 155 | 0 (0-2.35) | 509 | 0.79 (0.21-2.00) | 1996 | 0.80 (0.46-1.30) |
| Mercapto mix^(i)^ | 1.0 | 0 |  | 0 |  | 0 |  | 565 | 0.18 (0-0.98) |
| Mercapto mix^(ii)^ | 2.0 | 2525 | 0.28 (0.11-0.57) | 501 | 0.40 (0.05-1.43) | 0 |  | 1186 | 0.76 (0.35-1.44) |
| Mercaptobenzothiazole | 2.0 | 2631 | 0.15 (0.04-0.39) | 501 | 0 (0-0.73) | 509 | 0.39 (0.05-1.41) | 1996 | 0.65 (0.35-1.11) |
| **Resins/glues** |  |  |  |  |  |  |  |  |  |
| Colophonium | 20 | 2593 | 1.31 (0.91-1.83) | 501 | 3.99 (2.46-6.10) | 509 | 2.55 (1.37-4.33) | 1996 | 1.40 (0.93-2.02) |
| Epoxy resin | 1.0 | 2560 | 0.43 (0.21-0.77) | 501 | 1.20 (0.44-2.59) | 509 | 0.39 (0.05-1.41) | 1996 | 0.35 (0.14-0.72) |
| *p*-*tert*-Butylphenol formaldehyde resin | 1.0 | 2571 | 0.62 (0.36-1.01) | 501 | 1.00 (0.32-2.31) | 509 | 0.98 (0.32-2.28) | 2020 | 0.74 (0.42-1.22) |
| 2-Hydroxyethyl methacrylate | 2.0 | 3802 | 4.73 (4.08-5.46) | 501 | 6.39 (4.41-8.90) | 509 | 2.95 (1.66-4.81) | 721 | 3.88 (2.60-5.56) |
| **Other** |  |  |  |  |  |  |  |  |  |
| *p*-Phenylenediamine | 1.0 | 2622 | 3.85  (3.15-4.66) | 501 | 5.59 (3.75-7.98) | 509 | 5.30 (3.52-7.62) | 1996 | 5.51 (4.55-6.60) |
| Sesquiterpene lactone mix | 0.1 | 4416 | 0.20 (0.09-0.39) | 501 | 0.80 (0.22-2.03) | 509 | 0.20 (0-1.09) | 0 |  |
| Propolis | 10 | 3255 | 3.66 (3.04-4.36) | 501 | 16.4 (13.2-19.9) | 509 | 5.50 (3.69-7.85) | 87 | 3.45 (0.72-9.75) |
| Textile dye mix | 6.6 | 3803 | 3.13 (2.6-3.73) | 501 | 8.18 (5.94-10.9) | 0 |  | 807 | 3.59 (2.42-5.12) |

ES, Spain; GR, Greece; HU, Hungary; IT, Italy; epoxy resin, diglycidyl ether of bisphenol A; HICC, hydroxyisohexyl 3-cyclohexene carboxaldehyde; mercapto mix^(i)^, containing N-cyclohexylbenzothiazyl sulfenamide, dibenzothiazyl disulfide, and morpholinylmercaptobenzothiazole; mercapto mix^(ii)^, containing N-cyclohexylbenzothiazyl sulfenamide, mercaptobenzothiazole, dibenzothiazyl disulfide, and morpholinylmercaptobenzothiazole; MCI, methylchloroisothiazolinone; MI, methylisothiazolinone.

| **Allergen** | **Conc.** | **LT tested** | **LT % pos. (95% CI)** | **NL tested** | **NL % pos. (95% CI)** | **PL tested** | **PL % pos. (95% CI)** |
| --- | --- | --- | --- | --- | --- | --- | --- |
| **Metals** |  |  |  |  |  |  |  |
| Potassium dichromate | 0.5 | 701 | 1.14 (0.49-2.24) | 1931 | 12.2 (10.7-13.7) | 85 | 2.35 (0.29-8.24) |
| Cobalt chloride | 1.0 | 701 | 2.85 (1.75-4.37) | 1905 | 11.7 (10.3-13.2) | 85 | 11.8 (5.8-20.6) |
| Nickel sulphate | 5.0 | 701 | 14.3 (11.8-17.1) | 1930 | 22.0 (20.2-23.9) | 85 | 24.7 (16.0-35.3) |
| **Fragrances** |  |  |  |  |  |  |  |
| Fragrance mix I | 8.0 | 701 | 1.14 (0.49-2.24) | 1941 | 10.8 (9.47-12.3) | 85 | 5.88 (1.94-13.2) |
| Fragrance mix II | 14 | 701 | 1.28 (0.59-2.42) | 1944 | 7.36 (6.23-8.61) | 85 | 4.71 (1.3-11.61) |
| HICC | 5.0 | 701 | 0.29 (0.03-1.03) | 536 | 1.31 (0.53-2.67) | 85 | 0 (0-4.25) |
| *Myroxylon pereirae* (balsam of Peru) | 25 | 701 | 1.43 (0.69-2.61) | 1944 | 9.67 (8.39-11.1) | 85 | 5.88 (1.94-13.2) |
| **Preservatives** |  |  |  |  |  |  |  |
| Formaldehyde | 1.0^a^ | 0 |  | 1410 | 1.91 (1.27-2.77) | 0 |  |
| Formaldehyde | 2.0^a^ | 701 | 1.57 (0.79-2.79) | 533 | 1.50 (0.65-2.94) | 85 | 3.53 (0.73-9.97) |
| MCI/MI 3:1 | 0.01^a^ | 0 |  | 539 | 2.60 (1.43-4.32) | 0 |  |
| MCI/MI 3:1 | 0.02^a^ | 701 | 2.00 (1.10-3.33) | 1937 | 13.5 (12.0-15.1) | 85 | 4.71 (1.30-11.6) |
| Methylisothiazolinone | 0.05^a^ | 0 |  | 3 | 0 (0-70.8) | 0 |  |
| Methylisothiazolinone | 0.20^a^ | 701 | 3.28 (2.09-4.88) | 3321 | 10.2 (9.14-11.2) | 85 | 0 (0-4.25) |
| Paraben mix | 16 | 701 | 0.29 (0.03-1.03) | 1943 | 1.7 (1.17-2.38) | 85 | 0 (0-4.25) |
| Quaternium-15 | 1.0 | 701 | 0.43 (0.09-1.25) | 1945 | 0.41 (0.18-0.81) | 85 | 1.18 (0.03-6.38) |
| Methyldibromo glutaronitrile | 0.3 | 0 |  | 1411 | 10.7 (9.14-12.4) | 0 |  |
| Methyldibromo glutaronitrile | 0.5 | 701 | 1 (0.4-2.05) | 533 | 6.19 (4.3-8.59) | 85 | 3.53 (0.73-9.97) |
| **Medicaments, excipients** |  |  |  |  |  |  |  |
| Caine mix III (Benzo-,Cincho-,Tetracaine) | 10 | 701 | 0.14 (0-0.79) | 1939 | 3.35 (2.6-4.25) | 85 | 4.71 (1.30-11.6) |
| Budesonide | 0.01 | 701 | 0.29 (0.03-1.03) | 1945 | 0.57 (0.28-1.01) | 85 | 0 (0-4.25) |
| Budesonide | 0.1 | 0 |  | 534 | 0.37 (0.05-1.35) | 0 |  |
| Tixocortol pivalate | 0.1 | 701 | 0.29 (0.03-1.03) | 1940 | 0.67 (0.36-1.14) | 85 | 1.18 (0.03-6.38) |
| Tixocortol pivalate | 1.0 | 0 |  | 1395 | 0.57 (0.25-1.13) | 0 |  |
| Neomycin sulphate | 20 | 701 | 0.43 (0.09-1.25) | 1943 | 0.87 (0.51-1.4) | 85 | 4.71 (1.3-11.61) |
| Lanolin (wool alcohols) | 30 | 701 | 0.29 (0.03-1.03) | 1939 | 2.73 (2.05-3.56) | 85 | 4.71 (1.3-11.6) |
| **Rubber additives** |  |  |  |  |  |  |  |
| Thiuram mix | 1.0 | 701 | 0.43’ (0.09-1.25) | 1944 | 1.80 (1.26-2.5) | 85 | 3.53 (0.73-9.97) |
| *N*-Isopropyl-*N*’-phenyl-*p*-phenylenediamine | 0.1 | 701 | 0.29 (0.03-1.03) | 1943 | 0.72 (0.39-1.21) | 85 | 8.24 (3.38-16.23) |
| Mercapto mix^(i)^ | 1.0 | 0 |  | 1405 | 0.78 (0.39-1.4) | 0 |  |
| Mercapto mix^(ii)^ | 2.0 | 0 |  | 538 | 0 (0-0.68) | 85 | 0 (0-4.25) |
| Mercaptobenzothiazole | 2.0 | 701 | 0.43 (0.09-1.25) | 1945 | 0.57 (0.28-1.01) | 85 | 0 (0-4.25) |
| **Resins/glues** |  |  |  |  |  |  |  |
| Colophonium | 20 | 701 | 2.43 (1.42-3.85) | 1943 | 3.55 (2.77-4.47) | 85 | 4.71 (1.3-11.6) |
| Epoxy resin | 1.0 | 701 | 1.14 (0.49-2.24) | 529 | 3.59 (2.18-5.55) | 85 | 0 (0-4.25) |
| *p*-*tert*-Butylphenol formaldehyde resin | 1.0 | 701 | 0 (0-0.52) | 1945 | 0.82 (0.47-1.33) | 85 | 0 (0-4.25) |
| 2-Hydroxyethyl methacrylate | 2.0 | 701 | 1.85 (0.99-3.15) | 1939 | 3.61 (2.82-4.54) | 85 | 9.41 (4.15-17.7) |
| **Other** |  |  |  |  |  |  |  |
| *p*-Phenylenediamine | 1.0 | 701 | 1.14 (0.49-2.24) | 1931 | 3.31 (2.56-4.21) | 85 | 0 (0-4.25) |
| Sesquiterpene lactone mix | 0.1 | 701 | 0.14 (0-0.79) | 1887 | 1.64 (1.12-2.32) | 85 | 1.18 (0.03-6.38) |
| Propolis | 10 | 391 | 0.77 (0.16-2.23) | 1938 | 12.5 (11.1-14.0) | 85 | 1.18 (0.03-6.38) |
| Textile dye mix | 6.6 | 701 | 1.85 (0.99-3.15) | 1911 | 3.98 (3.15-4.95) | 84 | 14.3 (7.61-23.6) |

LT, Lithuania; NL, The Netherlands; PL, Poland; epoxy resin, diglycidyl ether of bisphenol A; HICC, hydroxyisohexyl 3-cyclohexene carboxaldehyde; mercapto mix^(i)^, containing N-cyclohexylbenzothiazyl sulfenamide, dibenzothiazyl disulfide, and morpholinylmercaptobenzothiazole; mercapto mix^(ii)^, containing N-cyclohexylbenzothiazyl sulfenamide, mercaptobenzothiazole, dibenzothiazyl disulfide, and morpholinylmercaptobenzothiazole; MCI, methylchloroisothiazolinone; MI, methylisothiazolinone.

| **Allergen** | **Conc.** | **PT tested** | **PT pos (95% CI)** | **SI tested** | **SI pos (95% CI)** | **UK tested** | **UK % pos. (95% CI)** |
| --- | --- | --- | --- | --- | --- | --- | --- |
| **Metals** |  |  |  |  |  |  |  |
| Potassium dichromate | 0.5 | 523 | 4.21 (2.65-6.30) | 1493 | 3.22 (2.38-4.24) | 2320 | 0.86 (0.53-1.33) |
| Cobalt chloride | 1.0 | 524 | 5.73 (3.90-8.07) | 1493 | 3.62 (2.73-4.69) | 2320 | 5.43 (4.54-6.43) |
| Nickel sulphate | 5.0 | 524 | 23.3 (19.7-27.1) | 1493 | 14.2 (12.5-16.1) | 2318 | 14.5 (13.0-16.0) |
| **Fragrances** |  |  |  |  |  |  |  |
| Fragrance mix I | 8.0 | 524 | 8.40 (6.17-11.1) | 1493 | 5.22 (4.15-6.48) | 2319 | 5.56 (4.66-6.57) |
| Fragrance mix II | 14 | 523 | 4.97 (3.27-7.20) | 1493 | 2.81 (2.03-3.78) | 2320 | 2.33 (1.75-3.03) |
| HICC | 5.0 | 522 | 0.96 (0.31-2.22) | 1493 | 0.80 (0.42-1.40) | 2320 | 0.73 (0.43-1.17) |
| *Myroxylon pereirae* (balsam of Peru) | 25 | 523 | 4.97 (3.27-7.2) | 1493 | 6.63 (5.42-8.01) | 2317 | 4.96 (4.11-5.93) |
| **Preservatives** |  |  |  |  |  |  |  |
| Formaldehyde | 1.0^a^ | 0 |  | 1493 | 1.47 (0.93-2.22) | 0 |  |
| Formaldehyde | 2.0^a^ | 523 | 0.57 (0.12-1.67) | 0 |  | 2320 | 0.95 (0.6-1.43) |
| MCI/MI 3:1 | 0.01^a^ | 0 |  | 1493 | 3.48 (2.61-4.54) | 0 |  |
| MCI/MI 3:1 | 0.02^a^ | 524 | 4.20 (2.65-6.29) | 0 |  | 2320 | 2.46 (1.87-3.17) |
| Methylisothiazolinone | 0.05^a^ | 0 |  | 0 |  | 0 |  |
| Methylisothiazolinone | 0.20^a^ | 523 | 4.02 (2.5-6.07) | 1493 | 3.55 (2.67-4.62) | 2320 | 2.24 (1.68-2.93) |
| Paraben mix | 16 | 0 |  | 1493 | 0.67 (0.32-1.23) | 456 | 0.44 (0.05-1.58) |
| Quaternium-15 | 1.0 | 521 | 0.19 (0-1.06) | 1493 | 0.47 (0.19-0.96) | 2320 | 0.39 (0.18-0.74) |
| Methyldibromo glutaronitrile | 0.3 | 0 |  | 0 |  | 2320 | 1.42 (0.98-1.99) |
| Methyldibromo glutaronitrile | 0.5 | 252 | 0 (0-1.45) | 1493 | 9.18 (7.76-10.8) | 0 |  |
| **Medicaments, excipients** |  |  |  |  |  |  |  |
| Caine mix III (Benzo-,Cincho-,Tetracaine) | 10 | 521 | 2.30 (1.20-3.99) | 0 |  | 2320 | 0.56 (0.30-0.96) |
| Budesonide | 0.01 | 523 | 0.57 (0.12-1.67) | 1493 | 1.61 (1.03-2.38) | 0 |  |
| Budesonide | 0.1 | 0 |  | 0 |  | 2320 | 0.04 (0-0.24) |
| Tixocortol pivalate | 0.1 | 0 |  | 1493 | 0.47 (0.19-0.96) | 0 |  |
| Tixocortol pivalate | 1.0 | 0 |  | 0 |  | 2320 | 0.26 (0.09-0.56) |
| Neomycin sulphate | 20 | 523 | 0.38 (0.05-1.37) | 1493 | 1.27 (0.77-1.98) | 2319 | 0.34 (0.15-0.68) |
| Lanolin (wool alcohols) | 30 | 523 | 2.10 (1.05-3.73) | 1493 | 1.00 (0.56-1.65) | 2320 | 0.43 (0.21-0.79) |
| **Rubber additives** |  |  |  |  |  |  |  |
| Thiuram mix | 1.0 | 524 | 2.86 (1.61-4.68) | 1493 | 2.34 (1.64-3.25) | 2320 | 1.38 (0.95-1.94) |
| *N*-Isopropyl-*N*’-phenyl-*p*-phenylenediamine | 0.1 | 522 | 0.57 (0.12-1.67) | 1493 | 0 (0-0.25) | 2320 | 0.17 (0.05-0.44) |
| Mercapto mix^(i)^ | 1.0 | 0 |  | 0 |  | 0 |  |
| Mercapto mix^(ii)^ | 2.0 | 523 | 0.38 (0.05-1.37) | 1493 | 0.40 (0.15-0.87) | 2057 | 0.05 (0-0.27) |
| Mercaptobenzothiazole | 2.0 | 523 | 0.38 (0.05-1.37) | 1493 | 0.40 (0.15-0.87) | 2320 | 0.17 (0.05-0.44) |
| **Resins/glues** |  |  |  |  |  |  |  |
| Colophonium | 20 | 524 | 1.91 (0.92-3.48) | 1493 | 4.09 (3.14-5.22) | 2320 | 2.20 (1.64-2.88) |
| Epoxy resin | 1.0 | 521 | 1.15 (0.42-2.49) | 1493 | 1.41 (0.87-2.14) | 2320 | 0.69 (0.39-1.12) |
| *p*-*tert*-Butylphenol formaldehyde resin | 1.0 | 522 | 0.19 (0-1.06) | 1493 | 0.33 (0.11-0.78) | 2320 | 0.34 (0.15-0.68) |
| 2-Hydroxyethyl methacrylate | 2.0 | 522 | 4.79 (3.12-6.99) | 1493 | 1.34 (0.82-2.06) | 2308 | 3.12 (2.45-3.91) |
| **Other** |  |  |  |  |  |  |  |
| *p*-Phenylenediamine | 1.0 | 521 | 2.88 (1.62-4.70) | 1493 | 2.75 (1.98-3.71) | 2313 | 3.63 (2.91-4.48) |
| Sesquiterpene lactone mix | 0.1 | 0 |  | 1493 | 0.80 (0.42-1.40) | 2320 | 0.82 (0.49-1.28) |
| Propolis | 10 | 252 | 0.79 (0.10-2.84) | 1493 | 1.61 (1.03-2.38) | 1864 | 0.86 (0.49-1.39) |
| Textile dye mix | 6.6 | 522 | 2.68 (1.47-4.46) | 1493 | 4.02 (3.08-5.14) | 0 |  |

PT, Portugal; SI, Slovenia; UK, United Kingdom; Epoxy resin, diglycidyl ether of bisphenol A; HICC, hydroxyisohexyl 3-cyclohexene carboxaldehyde; mercapto mix^(i)^, containing N-cyclohexylbenzothiazyl sulfenamide, dibenzothiazyl disulfide, and morpholinylmercaptobenzothiazole; mercapto mix^(ii)^, containing N-cyclohexylbenzothiazyl sulfenamide, mercaptobenzothiazole, dibenzothiazyl disulfide, and morpholinylmercaptobenzothiazole; MCI, methylchloroisothiazolinone; MI, methylisothiazolinone.
